# Fatty Acid Profiling and Chemometric Analyses for *Zanthoxylum* Pericarps from Different Geographic Origin and Genotype

**DOI:** 10.3390/foods9111676

**Published:** 2020-11-16

**Authors:** Yao Ma, Jieyun Tian, Xiaona Wang, Chen Huang, Mingjing Tian, Anzhi Wei

**Affiliations:** 1 College of Forestry, Northwest A&F University, Yangling 712100, China; mayao277000@nwafu.edu.cn (Y.M.); tianjieyun@nwafu.edu.cn (J.T.); 17709590958@nwafu.edu.cn (X.W.); hc19990513@nwafu.edu.cn (C.H.); 15531971892@nwafu.edu.cn (M.T.); 2 Research Centre for Engineering and Technology of Zanthoxylum, State Forestry Administration, Yangling 712100, China

**Keywords:** fatty acids of *Zanthoxylum* pericarps, geographic influence factors on fatty acid composition, chemometrics based on fatty acid compositions

## Abstract

*Zanthoxylum* plants, important aromatic plants, have attracted considerable attention in the food, pharmacological, and industrial fields because of their potential health benefits, and they are easily accessible because of the wild distribution in most parts of China. The chemical components vary with inter and intraspecific variations, ontogenic variations, and climate and soil conditions in compositions and contents. To classify the relationships between different *Zanthoxylum* species and to determine the key factors that influence geographical variations in the main components of the plant, the fatty acid composition and content of 72 pericarp samples from 12 cultivation regions were measured and evaluated. Four fatty acids, palmitic acid (21.33–125.03 mg/g), oleic acid (10.66–181.37 mg/g), linoleic acid (21.98–305.32 mg/g), and linolenic acid (0.06–218.84 mg/g), were the most common fatty acid components in the *Zanthoxylum* pericarps. Fatty acid profiling of *Zanthoxylum* pericarps was significantly affected by *Zanthoxylum* species and geographical variations. Stearic acid and oleic acid in pericarps were typical fatty acids that distinguished *Zanthoxylum* species based on the result of discriminant analysis (DA). Palmitic acid, palmitoleic acid, trans-13-oleic acid, and linoleic acid were important differential indicators in distinguishing given *Zanthoxylum* pericarps based on the result of orthogonal partial least squares discriminant analysis (OPLS-DA). In different *Zanthoxylum* species, the geographical influence on fatty acid variations was diverse. This study provides information on how to classify the *Zanthoxylum* species based on pericarp fatty acid compositions and determines the key fatty acids used to classify the *Zanthoxylum* species.

## 1. Introduction

Prickly ash (*Zanthoxylum*) and its relative species are widely cultivated in some Southeast Asian countries, especially in China [1,2]. Most of these *Zanthoxylum* species contain several types of bioactive components, including volatile oils [3,4,5], flavonoids and polyphenols [6], and acid amide compounds [7,8]. Two notable species are *Z. armatum* DC. (ZA) and *Z. bungeanum* Maxim. (ZB). The *Zanthoxylum* species with green pericarps is commonly known as Green Huajiao, while the species with red pericarps is commonly known as Red Huajiao. These popular *Zanthoxylum* species are used as spices and have potential applications in therapeutic medicines, cosmetics, and other industries [9,10]. The planting area of 1,670,000 hectares and output of 350,000 tons of *Zanthoxylum* species in China are both the largest in the world, producing enormous economic value of more than 40 billion dollars [11]. The distribution of these *Zanthoxylum* species overlaps in China, and the pericarps within the same species from different producing regions are similar in the phenotypic characteristics [12]. Contents and compositions of the bioactive components in the plants are easily affected by tree species, breeding, management measures, and climate and soil conditions [1,11,13]. Thus, the phytochemical profiling of pericarps from different geographic origins of *Zanthoxylum* and from different *Zanthoxylum* species needs to be determined.

Fatty acids are one of the basic components required by organisms to survive [14,15]. On the one hand, fatty acids have important physiological functions and are the main precursors for synthesizing some signal molecules and aroma esters [16]. On the other hand, fatty acids can prevent mechanical damage and heat emission together with other substances [17,18]. Further, fatty acids are important nutrients that can lower blood pressure, increase lipoprotein and apolipoprotein levels, and reduce body weight [19,20,21]. However, some fatty acids cannot be synthesized by humans and can only be obtained from food. The intake of irrational amounts and ratios of fatty acids causes some diseases [22]. Moreover, fatty acid compositions coupled with chemometrics have been used as a biological method for species and origin identification of some plants [23,24]. Therefore, the determination of fatty acids from different *Zanthoxylum* pericarps is feasible and necessary.

First, fatty acid profiling of *Zanthoxylum* pericarps from different geographic origins and from different genotypes were determined using gas-phase mass spectrometry. Several chemometric methods based on the fatty acid date were used to classify different *Zanthoxylum* species and determine the key differential fatty acids between pericarps from different species. Moreover, redundancy analysis (RDA) was applied to determine the relationships between geographic factors (location, climate, and soil conditions) and the fatty acid compositions in pericarps for the widely cultivated *Zanthoxylum* species (ZA and ZB). This study identifies fatty acid variations among different *Zanthoxylum* species from different geographic origins and determines the key factors that influence fatty acid composition in pericarps. Moreover, the results are potentially relevant to quality assessments of *Zanthoxylum* pericarps.

## 2. Materials and Methods

### 2.1. Sample Collection and Preparation

A total of 72 *Zanthoxylum* pericarp samples were collected from 12 provinces in China (Shandong, Hebei, Shanxi, Shaanxi, Henan, Gansu, Qinghai, Sichuan, Chongqing, Guizhou, Jiangxi, and Yunnan) in 2018. In total, 55 samples were red pericarps (Z1-Z55) and 17 samples were green pericarps (Z56-Z72). These pericarp samples were divided into four groups based on the cladogram of ITS2 sequencing in our previous research: ZA is *Z. armatum*; ZB1 samples, belonging to *Z. bungeanum*, are represented by pericarps from Hancheng; and ZB2 samples, belonging to *Z. bungeanum*, are represented by pericarps from Fengxian; Others refers to the rest of the samples whose pericarps are red but do not belong to *Z. bungeanum* [25].

The *Zanthoxylum* trees were in the full productive period (8–12 years old). About 5 kg of pericarp samples were collected at random from five trees at each plantation and then mixed. About 500 g of topsoil (0–5 cm) were obtained using an X-shaped sampling grid at each plantation. Samples from each plantation were separated into three replicates and detailed information was recorded. Then, all the samples were sealed in valve bags and transported to the laboratory. The soil samples and pericarp samples were each dried and ground to a homogenized powder.

### 2.2. Determination of Environmental Factors

The location, longitude (Long), latitude (Lat), and altitude (Alt) datasets were collected using a GPS real-time altitude app developed by Fuzhou lexun network technology co., LTD, China; the closest-proximity values of climate, mean atmospheric pressure (AtP), mean temperature (MT), mean relative humidity (MRH), and mean annual precipitation (MAP) were obtained from the National Meteorological Inform Centre (http://data.cma.cn/). The organic matter contents in soils (OM) were detected using the potassium dichromate concentrated sulphuric acid external heating method. A Sartorius PB-10 pH meter (Sartorius AG, Goettingen, Germany) was used to determine values of soil pH. An AA3 Auto Analyzer (SEAL, Germany) was used to determine the contents of soil total nitrogen (N_t_) after digestion using concentrated sulfuric acid (5 mL) combined with sodium nitroprusside as a catalyst. The alkaline hydrolysis diffusion method was used to determine the contents of available nitrogen (N_a_). The molybdenum-antimony resistance colorimetric method was used to determine the contents of total phosphorus (P_t_) after sodium hydroxide melting, while the molybdenum-antimony colorimetric method was used to determine the contents of available phosphorus (P_a_) after sodium bicarbonate extraction. Flame photometry was used to determine the contents of total potassium (K_t_) after sodium hydroxide melting and to determine the contents of available potassium (K_a_) after ammonium acetate leaching. An inductively coupled plasma-optical emission spectrometry (PerkinElmer Co., Waltham, MA, USA) was used to determine the contents of aluminum (Al), cadmium (Cd), lead (Pb), manganese (Mn), and nickel (Ni) in the soil samples, and an AFS-2100 atomic fluorescence spectrophotometer (Beijing Haiguang Instrument Co. Ltd., Beijing, China) was used to determine the arsenic (As) content in the soil samples after wet-digesting the samples with mixed acids. This information was published in our previous study [11,25].

### 2.3. Fatty Acid Extraction and Analysis

A mixture solvent (2 chloroform and 1methanol) was used to extract fatty acids [26]. The extraction of pericarps from each plantation was repeated three times. Then, methyl esterification was performed with the mixture solvent (9 methanol and 1sulfuric acid) [27]. Then, 3 mL of methyl tridecanoate (dissolved with n-hexane to 0.1 mg/mL) were used to extract fatty acid methyl esters and Thermo Scientific Trace 1310 gas chromatograph (Thermo Fisher Scientific Inc., Waltham, MA, USA) equipped with a flame ionization detector system was used to determine the fatty acid compositions.

The carrier gas was helium gas (a purity of 99.99%) and the carrier flow was 1 mL/min with a split flow of 20 mL/min. The initial temperature was 80 °C and maintained for 1 min, and then gradually increased to 175 °C with a rate of 50 °C/min. The temperature was increased to 200 °C at a rate of 5 °C/min after 1 min at 175 °C, Then, it was maintained at 200 °C for 1 min and further increased to 210 °C with a rate of 2 °C/min. At last, the temperature was increased to 230 °C with a rate of 5 °C/min and held for 10 min. The qualitative analyses of fatty acids compared their mass spectra fragmentation with the National Institute of Standards and Technology and compared the retention times of authentic methyl esters mixture standards (C4-C24, Shanghai yuanye Bio-Technology Co., Ltd., Shanghai, China). Additionally, quantification of the fatty acids was carried out using the external standard method using the corresponding program based on peak area (Table S1). The content of each fatty acid was described as milligrams of per g pericarps (mg/g).

### 2.4. Data Analyses

The mean, median, standard deviation, variance, skewness coefficient, *p* value for the Kolmogorov–Smirnov normality test, and histogram were used to assess whether the data came from a normally distributed population (Figure S1). The data was Z-score transformed prior to the statistical analyses (Tables S2–S4). Box plot, cluster heat map (CHM), principal component analysis (PCA), and discriminant analysis (DA) were performed by OriginPro 2018C (Originlab, Northampton, USA). Orthogonal partial least squares discriminant analysis (OPLS-DA) was performed using the online software (https://www.omicshare.com/tools/Home/Soft/getsoft). RDA was performed using the Canoco 5 program.

## 3. Results and Discussion

### 3.1. The Fatty Acid Profiling in Pericarps among Different Zanthoxylum Species

Fatty acid profiling is a good indicator of the quality and stability of oil. Therefore, determination of fatty acid profiling is necessary [28]. As shown in Figure S2, a total of 10 main fatty acids were detected in the pericarp samples from four *Zanthoxylum* species. The most common fatty acid components in the *Zanthoxylum* pericarp samples were palmitic acid (C16:0, 39.89 mg/g for ZA, 43.25 mg/g for ZB1, 34.41 mg/g for ZB2, and 35.28 mg/g for other species), palmitoleic acid (C16:1, 90.98 mg/g for ZA, 32.93 mg/g for ZB1, 8.87 mg/g for ZB2, and 17.85 mg/g for other species), oleic acid (C18:1n9, 21.81 mg/g for ZA, 52.59 mg/g for ZB1, 36.16 mg/g for ZB2, and 43.29 mg/g for other species), linoleic acid (C18:2, 39.37 mg/g for ZA, 56.99 mg/g for ZB1, 32.86 mg/g for ZB2, and 49.08 mg/g for other species), and linolenic acid (C18:3, 95.64 mg/g for ZA, 75.59 mg/g for ZB1, 66.23 mg/g for ZB2, and 70.05 mg/g for other species). Similar results were also observed in *Zanthoxylum* seeds [29]. It was worth noting that the presence of C16:1 was higher in ZA pericarps, indicating that ZA pericarps were the potential exploit source of C16:1.

**Figure 1:**
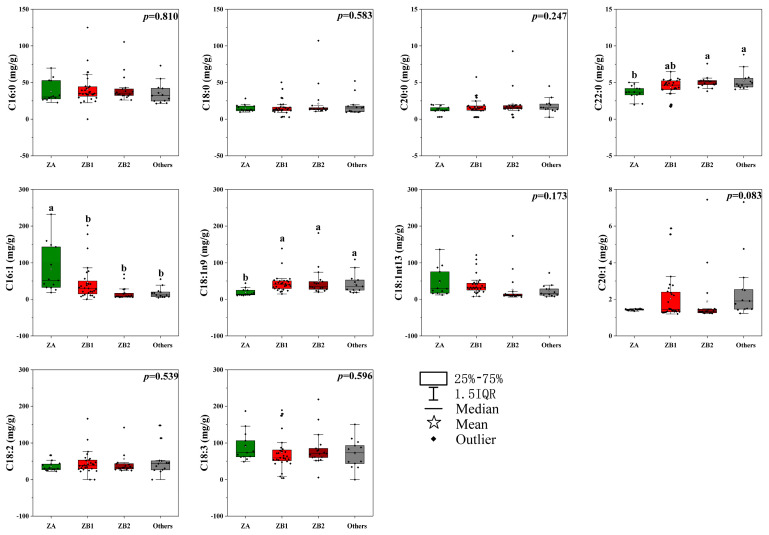
The fatty acid content and composition in pericarps among different *Zanthoxylum* groups. The different letters (a, b, or ab) above the histogram indicate significant differences among the *Zanthoxylum* species, and no letters above the histogram means no significant difference among different *Zanthoxylum* species (*p* < 0.01); ZA is *Z. armatum*; ZB1 samples, belonging to *Z. bungeanum*, are represented by pericarps from Hancheng; ZB2 samples, belonging to *Z. bungeanum*, are represented by pericarps from Fengxian; others refers to the rest of the samples whose pericarps are red but do not belong to *Z. bungeanum*; C16:0 is palmitic acid; C18:0 is stearic acid; C20:0 is eicosanoic acid; C22:0 is behenic acid; C16:1 is palmitoleic acid; C18:1n9 is oleic acid; C18:1n13t is trans-13-oleic acid; C20:1 is cis-11-eicosenoic acid; C18:2 is linoleic acid; C18:3 is linolenic acid; and IQR is interquartile range.

The comparative analysis of 10 fatty acids in the pericarps from 4 groups is shown in Figure 1. The content differences of C16:1, C18:1n9, and behenic acid (C22:0) among the four species were significant. The ZA pericarps had the highest content of C16:1 (18.07–232.46 mg/g) and the lowest content of C18:1n9 (10.66–44.92 mg/g) and C22:0 (1.98–5.41 mg/g). Genotype and environment were the main factors influencing how plants respond to changes in the environment and that affect the production of fatty acids, which often increases due to different biotic and abiotic stresses [30]. The *Zanthoxylum* species and plantation in our study had remarkable influences on the fatty acid content and composition of pericarps. The fatty acids in the same species from different plantations varied significantly, and the contents of fatty acids from some plantations were abnormal. Thus, the detailed relationship between fatty acid content and environmental factors of these plantations should be explored.

**Figure 2:**
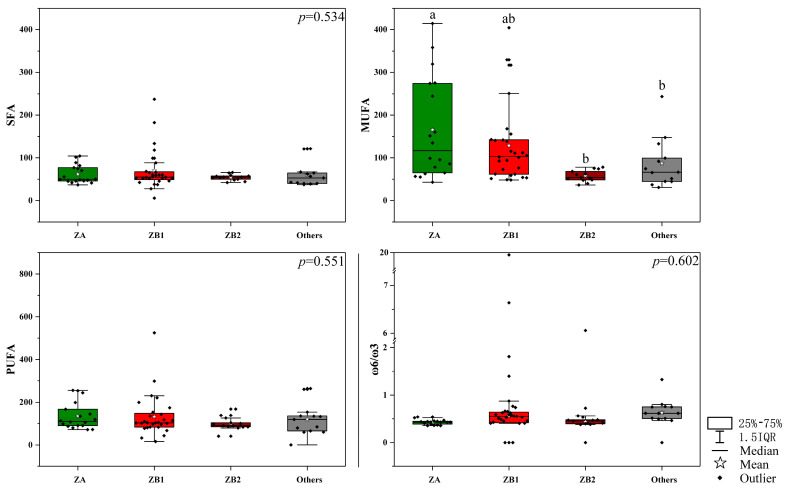
The total saturated fatty acid, monounsaturated fatty acid, polyunsaturated fatty acid content, and ω6/ω3 value in pericarps among different *Zanthoxylum* groups. The different letters (a, b, or ab) above the histogram indicate significant differences among the *Zanthoxylum* species, and no letters above the histogram means no significant difference among different *Zanthoxylum* species (*p* < 0.01); ZA is *Z. armatum*; ZB1 samples, belonging to *Z. bungeanum*, are represented by pericarps from Hancheng; ZB2 samples, belonging to *Z. bungeanum*, are represented by pericarps from Fengxian; Others refers to the rest of the samples whose pericarps are red but do not belong to *Z. bungeanum*; SFA is saturated fatty acid; MUFA is monounsaturated fatty acid; PUFA is polyunsaturated fatty acid; and IQR is interquartile range.

Due to the presence of various functional fatty acids, which are beneficial for human health, extraction and utilization of *Zanthoxylum* pericarp oils should be encouraged and implemented. As shown in Figure 2, the content difference of monounsaturated fatty acid in pericarps among the four groups was significant (165.21 mg/g for ZA, 128.88 mg/g for ZB1, 57.74 mg/g for ZB2, and 87.05 mg/g for other species), but the difference of the saturated fatty acid (61.56 mg/g for ZA, 70.42 mg/g for ZB1, 54.29 mg/g for ZB2, and 60.60 mg/g for other species) and polyunsaturated fatty acid (135.01 mg/g for ZA, 132.57 mg/g for ZB1, 99.09 mg/g for ZB2, and 119.13 mg/g for other species) was not significant (*p* > 0.01). Moreover, ω6 and ω3 have different functional properties as part of human diets and act in combination to regulate several human physiological processes [13]. Many diseases of animals and humans are related to the ratio of ω-6/ω-3 [31]. The ω6/ω3 ratio has frequently been used to analyze the nutritional oil and fat contents, with values lower than 4.0 being recommended by the UK Department of Healthy [32]. The values of ω-6/ω-3 ranged from 0.35 to 1.81, except for the abnormally high values (Z42, 19.96; Z43, 6.11; Z44, 6.72). These values of the ω-6/ω-3 ratio supported the healthy potential of the *Zanthoxylum* pericarps according to their fatty acid composition.

### 3.2. Chemometric Analyses for Zanthoxylum Pericarps Based on Fatty Acid Data

To classify *Zanthoxylum* species and determine the differences among pericarps from geographic origins, several classification methods were used based on the fatty acid data. Moreover, the relationships between geographic factors (location, climate, and soil conditions) and the fatty acid compositions in pericarps for the widely cultivated *Zanthoxylum* species were determined.

#### 3.2.1. Principal Component Analysis

**Figure 3:**
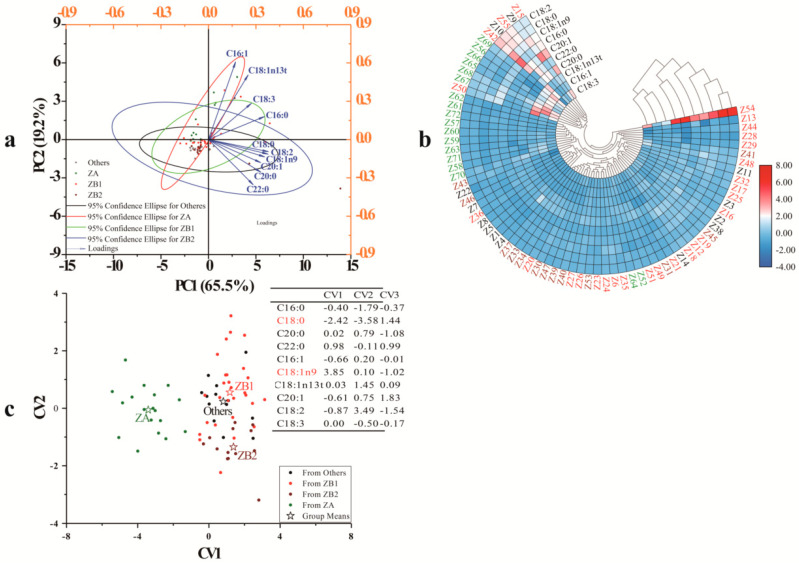
Geographical differentiation of *Zanthoxylum* pericarps from 72 plantations based on fatty acids compositions in pericarps: (**a**) principle component analysis of loading plot, (**b**) cluster heat map, (**c**) discriminant analysis. PC is principle component; CV is canonical variable; ZA is *Z. armatum*; ZB1 samples, belonging to *Z. bungeanum*, are represented by pericarps from Hancheng; ZB2 samples, belonging to *Z. bungeanum*, are represented by pericarps from Fengxian; Others refers to the rest of the samples whose pericarps are red but do not belong to *Z. bungeanum*; C16:0 is palmitic acid; C18:0 is stearic acid; C20:0 is eicosanoic acid; C22:0 is behenic acid; C16:1 is palmitoleic acid; C18:1n9 is oleic acid; C18:1n13t is trans-13-oleic acid; C20:1 is cis-11-eicosenoic acid; C18:2 is linoleic acid; and C18:3 is linolenic acid.

First, PCA was used to better understand the chemometric characteristics of the various groups (Figure 3a). The two principal components (PC1—65.5% and PC2—19.2%) accounted for 84.7% of the variation, with only a 15.3% loss of information. All the fatty acid components of the *Zanthoxylum* pericarp samples had positive loadings, which contributed to the variations of the pericarp samples in PC1; further, PC1 had high component loadings from C16:0, stearic acid (C18:0), C18:1n9, and C18:2. PC2 had high negative component loadings from C22:0 and positive component loadings from C16:1 and trans-13-oleic acid (C18:1n13t). The components with high component loadings contributed most to the sample classification. The sample distribution was not as similar as the species groups, indicating the dissimilarity in fatty acid compositions among pericarps from the same species but different geographic origins due to variations in environmental factors at the plantations.

#### 3.2.2. Cluster Heat Map (CHM)

CHM, an unsupervised pattern recognition method, was applied to classify the 72 *Zanthoxylum* pericarp samples based on the normalized value of the detected components in the pericarps (Figure 3b). Moreover, the content differences for each fatty acid in the pericarps from each geographic origin sample were also observed. C16:1, C18:1n9, and C18:3 shared a similar trend in the pericarps for all plantations, while the rest of the fatty acids shared a different trend. A similar fatty acid trend means a similar synthesis and accumulation pathway of fatty acids in the pericarps. In addition, three groups were identified: Z54 (Zhenfeng, Guizhou) was the sole member of one group, the second group contained 12 samples—Z9 (Pingshan, Hebei), Z10 (Wutai, Shanxi), Z15 (Ruicheng, Shanxi), Z42 (Gangu, Gansu), Z50 (Yiyuan, Sichuan), Z55 (Qixingguan, Guizhou), Z56 (Hanyuan, Sichuan), Z65 (Fengdu, Chongqing), Z66 (Yongshan, Sichuan), Z67(Ludian, Yunnan), Z68 (Qiaojiaxian, Yunnan), and Z69 (Qixingguan, Guizhou); most of the samples were mixed and classified into the third group. The clusters of samples from different plantations were consistent with the results of PCA (samples from different plantations were overlapped), which was not consistent with the ITS2 result. Consequently, factors that affect fatty acid synthesis processes need to be explored.

#### 3.2.3. Discriminant Analysis (DA)

A supervised analysis, DA, was used to better understand the categorization of the 72 samples (Figure 3c). Four distinct groups (ZA, ZB1, ZB2, and others) were generated according to the *Zanthoxylum* species. For the discrimination function, C18:0 and C18:1n9 in pericarps were typical fatty acids that distinguished four *Zanthoxylum* groups. The standard of discrimination was as follows: 10 fatty acids were substituted into two equations, and then the unknown samples were assigned to a group compared with the value means of the canonical variable (CV) of the training group date, CV1 (−3.41 for ZA, 1.18 for ZB1, 1.38 for ZB2, and 0.81 for others), CV2 (−0.06 for ZA, 0.55 for ZB1, −1.35 for ZB2, and 0.24 for others), and CV3 (−0.06 for ZA, −0.35 for ZB1, −0.13 for ZB2, and 0.97 for others). Cross-validation proved 19.44% of the error rate (0.00% for ZA, 39.29% for ZB1, 7.69% for ZB2, and 30.77% for others), indicating that the *Z. bungeanum* and other species could be misjudged.

#### 3.2.4. Orthogonal Partial Least Squares Discriminant Analysis

**Figure 4:**
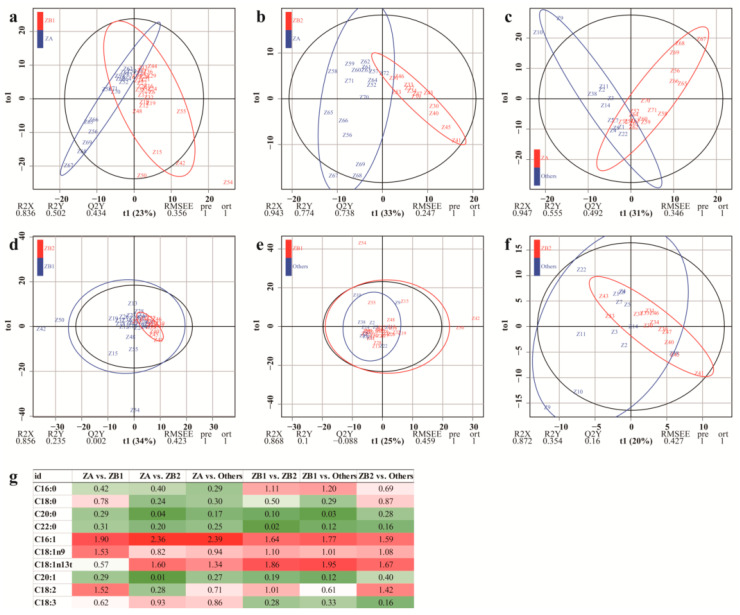
Orthogonal partial least squares discriminant analysis (OPLS-DA) between different *Zanthoxylum* species: (**a**), OPLS-DA between ZA and ZB1; (**b**), OPLS-DA between ZA and ZB2; (**c**), OPLS-DA between ZA and Others; (**d**), OPLS-DA between ZB1 and ZB2; (**e**), OPLS-DA between ZB1 and Others; (**f**), OPLS-DA between ZB2 and others; (**g**), Variable important in projection based on OPLS-DA between different *Zanthoxylum* species. ZA is *Z. armatum*; ZB1 samples, belonging to *Z. bungeanum*, are represented by pericarps from Hancheng; ZB2 samples, belonging to *Z. bungeanum*, are represented by pericarps from Fengxian; Others refers to the rest of the samples whose pericarps are red but do not belong to *Z. bungeanum*; C16:0 is palmitic acid; C18:0 is stearic acid; C20:0 is eicosanoic acid; C22:0 is behenic acid; C16:1 is palmitoleic acid; C18:1n9 is oleic acid; C18:1n13t is trans-13-oleic acid; C20:1 is cis-11-eicosenoic acid; C18:2 is linoleic acid; and C18:3 is linolenic acid.

The ZA and ZB species are widely cultivated and the fatty acid composition difference between the two species is significant. The pericarps from other species are similar to pericarps from ZB species in colors and the discrimination between two species is difficult. Thus, OPLS-DA was applied to determine the differences in fatty acid composition between different species (Figure 4). Variables important in projection (VIP) can measure the influence of intensity and the explanatory ability of each indicator accumulating difference on the discrimination of each group of samples. The indicators were important when the values of VIP were more than one [33]. In view of the values of these fatty acids (Figure 4g), C16:1 was an important fatty acid in distinguishing the pericarps from different species with the values of VIP > 1 (1.90, 2.36, 2.39, 1.64, 1.77, and 1.59 between different species); C18:1n13t was the second important indicator that distinguished ZA from ZB2 (1.60, Figure 4b), distinguished ZA from others (1.34, Figure 4c), distinguished ZB1 from ZB2 (1.86, Figure 4d), distinguished ZB1 from others (1.95, Figure 4e), and distinguished ZB2 from others (1.67, Figure 4f); C18:1n9 was the third important indicator that distinguished ZA from ZB1 (1.53, Figure 4a), distinguished ZB1 from ZB2 (1.10, Figure 4d), distinguished ZB1 from others (1.01, Figure 4e), and distinguished ZB2 from others (1.08, Figure 4f). Moreover, C18:2 could distinguish ZA from ZB1 (1.52, Figure 4a), distinguish ZB1 from ZB2 (1.01, Figure 4d), and distinguish ZB2 from others (1.42, Figure 4f); C16:0 could distinguish ZB1 from ZB2 (1.11, Figure 4d) and ZB1 from others (1.20, Figure 4e). The remaining fatty acids contributed less in terms of distinguishing the samples.

*Z. bungeanum* and some other species are botanically related and are often confused due to their similar morphological characteristics; the pericarp colors of these species are red when the fruit is ripe and marketed. However, greater variations in internal quality were found in samples from different plantations, including different acid amide components of pericarps [8], fatty acid of seeds [29], and fatty acid composition of pericarps in this study. Three chemometric methods (CHM, PCA, and DA) showed that the separate distribution of ZA species was distinct, and the distributions of ZB1, ZB2, and other species overlapped, indicating that distinguishing between ZB1, ZB2, and others was difficult due to the similarity in fatty acid compositions between these pericarps. C18:0 and C18:1n9 in pericarps were typical fatty acids that distinguished *Zanthoxylum* species based on the result of DA. The fatty acids C16:0, C16:1, C18:1n13t, C18:1n9, and C18:2 were important differential indicators in distinguishing given *Zanthoxylum* pericarps based on the result of OPLS-DA.

### 3.3. The Influences of Environmental Factors on Fatty Acid Content and Composition

Analyses immediately following collection in wild populations of medicinal and aromatic plants were likely affected by plant species that could have masked the true environmental variability [19,34]. The species of ZA, ZB1, ZB2, and others were distinguished prior to fatty acid extraction, minimizing the potential masking effect of plant species [25]. The ZA and ZB species are widely cultivated, and the attention of environmental factors’ influence on these species in the pericarp fatty acid composition was more worthy. The influence of environmental factors on the fatty acid composition of the ZA, ZB1, and ZB2 species was different according to the results of interactive forward selection (Table S5). Long, Alt, OM, K_t_, P_a_, K_a_, Al, Cd, Pb, and Mn, with higher values of contributions, were chosen as influence factors on the fatty acid composition of ZA pericarps; K_a_, Cd, Al, MAP, N_a_, N_t_, Ni, K_t_, OM, and Pb were chosen as influence factors for ZB1 pericarps; and K_a_, MRH, pH, Cd, Alt, MAP, Lat, AtP, K_t_, and OM were chosen as influence factors for ZB2 pericarps. Moreover, P_a_ (pseudo-F = 5.2, *p* = 0.012), Al (pseudo-F = 3.6, *p* = 0.012), Cd (pseudo-F = 11.9, *p* = 0.002), Pb (pseudo-F = 10.2, *p* = 0.002), and Mn (pseudo-F = 3.1, *p* = 0.048) were the key influence factors on the fatty acid composition of ZA pericarps; N_t_ (pseudo-F = 4.6, *p* = 0.016), N_a_ (pseudo-F = 4.1, *p* = 0.016), K_a_ (pseudo-F = 10.5, *p* = 0.004), and Cd (pseudo-F = 8.2, *p* = 0.032) were the key influence factors on the fatty acid composition of ZB1 pericarps; and K_t_ (pseudo-F = 4.2, *p* = 0.044) was the key influence factor on the fatty acid composition of ZB2 pericarps.

**Figure 5:**
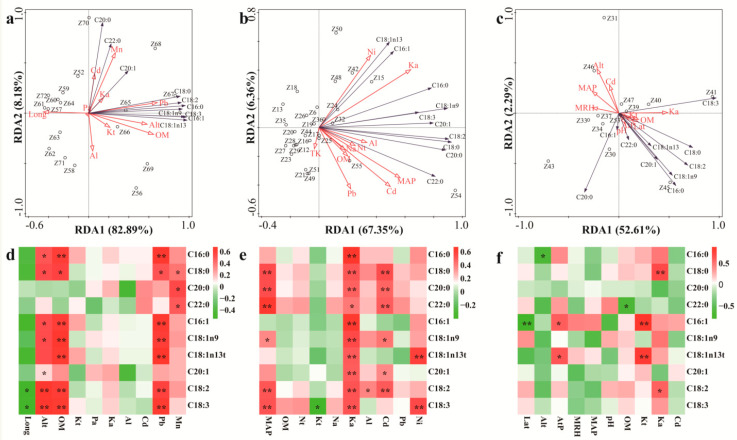
The relationships between environmental factors and pericarp fatty acid composition for different *Zanthoxylum* species: (**a**), the relationship between environmental factors and pericarp fatty acid composition for ZA; (**b**), the relationship between environmental factors and pericarp fatty acid composition for ZB1; (**c**), the relationship between environmental factors and pericarp fatty acid composition for ZB2; (**d**), the correlation between environmental factors and pericarp fatty acid composition for ZA; (**e**), the correlation between environmental factors and pericarp fatty acid composition for ZB1; (**f**), the correlation between environmental factors and pericarp fatty acid composition for ZB2. RDA is redundancy analyses; Long is longitude; Alt, altitude; AtP, atmospheric pressure; MT, mean temperature; MAP, mean annual precipitation; pH, power of hydrogen; OM, organic matter content in soil; Pt, total phosphorus content in soil; Kt, total potassium content in soil; Pa, available phosphorus content in soil; Ka, available potassium content in soil; Al, aluminium content in soil; Cd, cadmium content in soil; Pb, lead content in soil; Mn, manganese content in soil; C16:0, palmitic acid; C18:0, stearic acid; C20:0, eicosanoic acid; C22:0, behenic acid; C16:1, palmitoleic acid; C18:1n9, oleic acid; C18:1n13t, trans-13-oleic acid; C20:1, cis-11-eicosenoic acid; C18:2, linoleic acid; C18:3, and linolenic acid; * represents medium correlation (0.4–0.6); ** represents strong correlation (0.6–0.8).

In addition, RDA was used to analyze the relationships between the chosen environmental factors and fatty acid compositions of the ZA, ZB1, and ZB2 species. As shown in Figure 5, the chosen environmental variables explained 91.07% of the total variance in the fatty acid composition of the ZA species (RDA1- 82.89% and RDA2- 8.18%) and 73.71% of the total variance in fatty acid compositions of the ZB1 species (RDA1- 67.35% and RDA2- 6.36%), and they explained 54.90% of the total variance in fatty acid compositions of the ZB2 species (RDA1- 52.61% and RDA2- 2.29%). The model for ZB2 species with a lower explanation does not sufficiently explain the observed variation, and consequently, unknown factors could be involved. In contrast, the distribution of the ZA and ZB1 samples was dispersed, indicating that environmental factors in this model affect the fatty acid composition of the pericarps. The longer arrow lengths in Figure 5 indicate the factors are more important environmental factors, which cause the variations of fatty acids in pericarps from different geographical origins. Coupled to the correlation coefficient heat map, Alt, OM, and Pb were confirmed as the key environmental factors that cause the variations of fatty acids in pericarps for ZA (Figure 5d); MAP and K_a_ were key environmental factors that cause the variations of fatty acids in pericarps for ZB1 (Figure 5e); and K_t_ and K_a_ were key environmental factors that cause the variations of fatty acids in pericarps for ZB2 (Figure 5f). For ZA pericarps, Alt had significant positive effects on the content of C18:2 and C18:3; OM had significant positive effects on the content of C16:0, C16:1, C18:1n9, C18:1n13t, C18:2, and C18:3; and Pb had significant positive effects on the content of C16:0, C16:1, C18:1n9, C18:1n13t, C18:2, and C18:3 (Figure 5a,d). For ZB1 pericarps, MAP had significant positive effects on the content of C18:0, C20:0, C22:0, C18:2, and C18:3; and Ka had significant positive effects on the content of C16:0, C18:0, C20:0, C16:1, C18:1n9, C18:1n13t, C20:1, C18:2, and C18:3 (Figure 5b,e). The positive effects of MAP on C18:3 have been reported [35]. Moreover, low temperature was probably responsible for the high content of C18:3 [36]. For ZB2 pericarps, Kt had significant positive effects on the contents of C16:1 and C18:1n13t, and Ka had significant positive effects on the content of C18:0 (Figure 5c,f).

## 4. Conclusions

Ten main fatty acids in *Zanthoxylum* pericarps were identified. The most common fatty acid components in these *Zanthoxylum* samples were C16:0, C18:1n9, C18:2, and C18:3. Fatty acid profiling of *Zanthoxylum* pericarps from different species and different plantations varied significantly. A series of discriminative measures for *Zanthoxylum* pericarps based on fatty acids was proposed: the pericarps from ZA species were first to distinguish from all species using DA with the key fatty acids C18:0 and C18:1n9; then, the pericarps from ZB1, ZB2, and others were distinguished using OPLS-DA with key fatty acids C18:1n13t, C18:1n9, and C18:2. Moreover, the fatty acid content in pericarps was significantly affected by the type of *Zanthoxylum* species and environmental factors (Alt, OM, and Pb for ZA; MAP and K_a_ for ZB1; K_t_ and K_a_ for ZB2). Further, the results of this study proved it was possible to use fatty acid characteristics to determine the major variations in the compositions of different *Zanthoxylum* species from different plantations.

## Supplementary Materials

The following materials are available online at https://www.mdpi.com/2304-8158/9/11/1676/s1, Table S1 Qualitative and quantitative determinations of each fatty acid y, peak area; x, the content of fatty acid. Table S2 Raw data and transformed (Z-Score) values of location and climate for the 72 pericarp samples ZA is *Z. armatum*; ZB1 samples, belonging to *Z. bungeanum*, are represented by pericarps from Hancheng; ZB2 samples, belonging to *Z. bungeanum*, are represented by pericarps from Fengxian; Others refers to the rest of the samples whose pericarps are red but do not belong to *Z. bungeanum*. Table S3 Raw data and transformed (Z-Score) values of soil characteristics in different *Zanthoxylum* plantations ZA is *Z. armatum*; ZB1 samples, belonging to *Z. bungeanum*, are represented by pericarps from Hancheng; ZB2 samples, belonging to *Z. bungeanum*, are represented by pericarps from Fengxian; Others refers to the rest of the samples whose pericarps are red but do not belong to *Z. bungeanum*. Table S4 Raw data and transformed (Z-Score) values of fatty acid date in different *Zanthoxylum* pericarps ZA is *Z. armatum*; ZB1 samples, belonging to *Z. bungeanum*, are represented by pericarps from Hancheng; ZB2 samples, belonging to *Z. bungeanum*, are represented by pericarps from Fengxian; Others refers to the rest of the samples whose pericarps are red but do not belong to *Z. bungeanum*; C16:0 is palmitic acid; C18:0 is stearic acid; C20:0 is eicosanoic acid; C22:0 is behenic acid; C16:1 is palmitoleic acid; C18:1n9 is oleic acid; C18:1n13t is trans-13- oleic acid; C20:1 is cis-11-eicosenoic acid; C18:2 is linoleic acid; C18:3 is linolenic acid. Table S5 The effect of environmental factors on fatty acid variation for *Zanthoxylum* pericarps ZA is *Z. armatum*; ZB1 samples, belonging to *Z. bungeanum*, are represented by pericarps from Hancheng; ZB2 samples, belonging to *Z. bungeanum*, are represented by pericarps from Fengxian; ** represents strong significant influence; * represents significant influence; Long is longitude; Lat is latitude; Alt is altitude; MT is mean temperature; MRH is mean relative humidity; MAP is mean annual precipitation; pH is power of hydrogen; OM is organic matter content in soil; N_t_ is total nitrogen content in soil; P_t_ is total phosphorus content in soil; K_t_ is total potassium content in soil; N_a_ is available nitrogen content in soil; P_a_ is available phosphorus content in soil; K_a_ is available potassium content in soil; Al is aluminium content in soil; As is arsenic content in soil; Cd is cadmium content in soil; Mn is manganese content in soil; Ni is nickel content in soil. Figure S1 The mean, median, standard deviation (SD), variance (CV), skewness coefficient, and *p* value for the Kolmogorov–Smirnov normality test of fatty acids (mg/g). SFA is saturated fatty acid; MUFA is monounsaturated fatty acid; PUFA is polyunsaturated fatty acid; C16:0 is palmitic acid; C18:0 is stearic acid; C20:0 is eicosanoic acid; C22:0 is behenic acid; C16:1 is palmitoleic acid; C18:1n9 is oleic acid; C18:1n13t is trans-13- oleic acid; C20:1 is cis-11-eicosenoic acid; C18:2 is linoleic acid; C18:3 is linolenic acid. Figure S2 The relative abundance of three fatty acids among different *Zanthoxylum* species. The peaks without labels were identified as trace components. ZA is *Z. armatum*; ZB1 samples, belonging to *Z. bungeanum*, are represented by pericarps from Hancheng; ZB2 samples, belonging to *Z. bungeanum*, are represented by pericarps from Fengxian; Others refers to the rest of the samples whose pericarps are red but do not belong to *Z. bungeanum*; RT, retention time; C16:0 is palmitic acid; C18:0 is stearic acid; C20:0 is eicosanoic acid; C22:0 is behenic acid; C16:1 is palmitoleic acid; C18:1n9 is oleic acid; C18:1n13t is trans-13- oleic acid; C20:1 is cis-11-eicosenoic acid; C18:2 is linoleic acid; C18:3 is linolenic acid.
