# Supplementary material for: Fatty Acid Profiling and Chemometric Analyses for Zanthoxylum Pericarps from Different Geographic Origin and Genotype
Source: Foods. 2020 Nov 16;9(11):1676. doi: 10.3390/foods9111676 (PMC7698129; doi:10.3390/foods9111676)
Supplement: Supplementary file 1 [file foods-09-01676-s001.zip › Supplementary Tables.doc]

Supplementary tables

Fatty Acid Profiling and Chemometric Analyses for Zanthoxylum Pericarps from Different Geographic Origin and Genotype

Yao Ma^1,2^, Jieyun Tian ^1,2^, Xiaona Wang ^1,2^, Chen Huang^1^, Mingjing Tian^1^, and Anzhi Wei^1,2*^

^1^	College of Forestry, Northwest A&F University; mayao277000@nwafu.edu.cn (Y. Ma), tianjieyun@nwafu.edu.cn (J. Tian), 17709590958@nwafu.edu.cn (X. Wang), hc19990513@nwafu.edu.cn (C. Huang), 15531971892@nwafu.edu.cn (M. Tian), and weianzhi@nwafu.edu.cn (A. Wei)

^2^	Research Centre for Engineering and Technology of *Zanthoxylum*, State Forestry Administration; mayao277000@nwafu.edu.cn (Y. Ma), tianjieyun@nwafu.edu.cn (J. Tian), 17709590958@nwafu.edu.cn (X. Wang), and weianzhi@nwafu.edu.cn (A. Wei)*****	Correspondence: weianzhi@126.com; Tel. 86-029-87082211; Yangling, 712100, China

Supplementary tables

**Table S1** Qualitative and quantitative determinations of each fatty acid

y, peak area; x, the content of fatty acid.

**Table S2** Raw data and transformed (Z-Score) values of location and climate for the 72 pericarp samples

ZA is *Z. armatum*; ZB1 samples, belonging to *Z. bungeanum*, are represented by pericarps from Hancheng; ZB2 samples, belonging to *Z. bungeanum*, are represented by pericarps from Fengxian; Others refers to the rest of the samples whose pericarps are red but do not belong to *Z. bungeanum*.

**Table S3** **Table S3** Raw data and transformed (Z-Score) values of soil characteristics in different *Zanthoxylum* plantations

ZA is *Z. armatum*; ZB1 samples, belonging to *Z. bungeanum*, are represented by pericarps from Hancheng; ZB2 samples, belonging to *Z. bungeanum*, are represented by pericarps from Fengxian; Others refers to the rest of the samples whose pericarps are red but do not belong to *Z. bungeanum*.

**Table S4** Raw data and transformed (Z-Score) values of fatty acid date in different *Zanthoxylum* pericarps

ZA is *Z. armatum*; ZB1 samples, belonging to *Z. bungeanum*, are represented by pericarps from Hancheng; ZB2 samples, belonging to *Z. bungeanum*, are represented by pericarps from Fengxian; Others refers to the rest of the samples whose pericarps are red but do not belong to *Z. bungeanum*; C16:0 is palmitic acid; C18:0 is stearic acid; C20:0 is eicosanoic acid; C22:0 is behenic acid; C16:1 is palmitoleic acid; C18:1n9 is oleic acid; C18:1n13t is trans-13- oleic acid; C20:1 is cis-11-eicosenoic acid; C18:2 is linoleic acid; C18:3 is linolenic acid.

**Table S5** The effect of environmental factors on fatty acid variation for *Zanthoxylum* pericarps

ZA is *Z. armatum*; ZB1 samples, belonging to *Z. bungeanum*, are represented by pericarps from Hancheng; ZB2 samples, belonging to *Z. bungeanum*, are represented by pericarps from Fengxian; ** represents strong significant influence; * represents significant influence; Long is longitude; Lat is latitude; Alt is altitude; MT is mean temperature; MRH is mean relative humidity; MAP is mean annual precipitation; pH is power of hydrogen; OM is organic matter content in soil; N_t_ is total nitrogen content in soil; P_t_ is total phosphorus content in soil; K_t_ is total potassium content in soil; N_a_ is available nitrogen content in soil; P_a_ is available phosphorus content in soil; K_a_ is available potassium content in soil; Al is aluminium content in soil; As is arsenic content in soil; Cd is cadmium content in soil; Mn is manganese content in soil; Ni is nickel content in soil.

**Table S1** Qualitative and quantitative determinations of each fatty acid

| **Fatty acids** | **Retention time** | **Regression equation** | **Regression coefficient** |
| --- | --- | --- | --- |
| Palmitic acid (C16:0) | 8.35 | y =2*107+7*109x | 0.9969 |
| Palmitoleic acid (C16:1) | 8.66 | y = 4*106+4*109x | 0.9984 |
| Stearic acid (C18:0) | 11.16 | y =-3*106+3*109x | 0.9985 |
| Oleic acid (C18:1n9) | 11.48 | y =1*107+7*109x | 0.9988 |
| Trans-13- oleic acid (C18:1n13t) | 11.58 | y =–8*105+3*109x | 0.9989 |
| Linoleic acid (C18:2) | 12.30 | y =5*106+6*109x | 0.9992 |
| Linolenic acid (C18:3) | 13.55 | y =-2*106+3*109x | 0.9992 |
| Eicosanoic acid (C20:0) | 15.06 | y =-1*107+4*109x | 0.9956 |
| Cis-11-eicosenoic acid (C20:1) | 15.50 | y =-5*106+4*109x | 0.9987 |
| Behenic acid (C22:0) | 19.64 | y =-7*106+4*109x | 0.9987 |

y, peak area; x, the content of fatty acid.

**Table S2** Raw data and transformed (Z-Score) values of location and climate for the 72 pericarp samples

| **Group** | **Code** | **Origin Province** | | **County** | | **Longitude** | **Latitude** | **Altitude** | **Atmospheric pressure** | **Mean**  **temperature** | **Mean relative humidity** | **Mean annual precipitation** |
| --- | --- | --- | --- | --- | --- | --- | --- | --- | --- | --- | --- | --- |
| Others | Z1 | Shandong | Yiyuan | | | 118.33 | 36.03 | 210 | 981.7 | 12.5 | 62 | 689.5 |
| Others | Z2 | Shandong | Laicheng | | | 117.66 | 36.20 | 192 | 990.2 | 13.3 | 62 | 695.3 |
| Others | Z3 | Shandong | Shanting | | | 117.59 | 35.06 | 195 | 1008.0 | 14.4 | 66 | 820.2 |
| Others | Z4 | Hebei | Laishui | | | 115.45 | 39.67 | 298 | 1013.2 | 12.2 | 62 | 531 |
| Others | Z5 | Hebei | Tangxian | | | 114.78 | 38.86 | 191 | 1008.9 | 12.7 | 65 | 508.1 |
| Others | Z7 | Hebei | Shexian | | | 113.66 | 36.55 | 433 | 961.8 | 12.8 | 61 | 535 |
| Others | Z8 | Hebei | Fuping | | | 114.20 | 38.85 | 274 | 983.4 | 12.6 | 56 | 572.1 |
| Others | Z9 | Hebei | Pingshan | | | 114.20 | 38.25 | 138 | 983.4 | 12.6 | 56 | 572.1 |
| Others | Z10 | Shanxi | Wutai | | | 113.26 | 38.73 | 1032 | 892.4 | 7.0 | 59 | 503.7 |
| Others | Z11 | Shanxi | Xiaoyi | | | 111.78 | 37.15 | 753 | 928.0 | 11.0 | 57 | 448.5 |
| Others | Z14 | Shanxi | Yangcheng | | | 112.41 | 35.49 | 628 | 940.8 | 12.0 | 61 | 578.4 |
| Others | Z22 | Shaanxi | Huanglong | | | 109.72 | 35.60 | 1138 | 893.8 | 9.0 | 63 | 566.2 |
| Others | Z38 | Gansu | Dongxiang | | | 103.24 | 35.74 | 1807 | 759.5 | 5.6 | 62 | 534.9 |
| ZB1 | Z6 | Hebei | Shexian | | | 113.66 | 36.55 | 433 | 961.8 | 12.8 | 61 | 535 |
| ZB1 | Z12 | Shanxi | Zaoning | | | 110.66 | 35.80 | 835 | 907.1 | 10.5 | 59 | 515 |
| ZB1 | Z13 | Shanxi | Pingshun | | | 113.65 | 36.36 | 578 | 893.6 | 9.4 | 59 | 553 |
| ZB1 | Z15 | Shanxi | Ruicheng | | | 110.69 | 34.69 | 504 | 957.9 | 13.0 | 67 | 505.3 |
| ZB1 | Z16 | Henan | Lingbao | | | 110.59 | 34.50 | 692 | 961.7 | 13.5 | 66 | 600.5 |
| ZB1 | Z17 | Henan | Hubin | | | 111.19 | 34.77 | 358 | 969.1 | 14.4 | 61 | 549.6 |
| ZB1 | Z18 | Henan | Linzhou | | | 113.94 | 36.26 | 396 | 980.8 | 13.2 | 66 | 648.9 |
| ZB1 | Z19 | Henan | Baofeng | | | 112.90 | 33.93 | 212 | 1000.7 | 14.6 | 69 | 747.2 |
| ZB1 | Z20 | Shaanxi | Yanchang | | | 110.41 | 36.51 | 968 | 924.6 | 10.4 | 62 | 480.8 |
| ZB1 | Z21 | Shaanxi | Yichuan | | | 110.37 | 35.94 | 793 | 921.0 | 10.3 | 61 | 516 |
| ZB1 | Z23 | Shaanxi | Hancheng | | | 110.47 | 35.55 | 452 | 935.0 | 12.1 | 64 | 535.4 |
| ZB1 | Z24 | Shaanxi | Heyang | | | 110.32 | 35.30 | 549 | 935.0 | 12.1 | 64 | 535.4 |
| ZB1 | Z25 | Shaanxi | Huazhou | | | 109.72 | 34.43 | 741 | 975.3 | 13.7 | 71 | 577.5 |
| ZB1 | Z26 | Shaanxi | Fuping | | | 109.12 | 34.82 | 526 | 961.6 | 12.1 | 68 | 636.5 |
| ZB1 | Z27 | Shaanxi | Yaozhou | | | 109.03 | 34.94 | 820 | 934.5 | 12.7 | 62 | 543.4 |
| ZB1 | Z28 | Shaanxi | Chunhua | | | 108.66 | 34.75 | 737 | 902.0 | 10.6 | 67 | 585.7 |
| ZB1 | Z29 | Shaanxi | Chunhua | | | 108.66 | 34.75 | 737 | 902.0 | 10.6 | 67 | 585.7 |
| ZB1 | Z32 | Shaanxi | Chencang | | | 106.45 | 34.53 | 915 | 946.1 | 13.5 | 66 | 645.8 |
| ZB1 | Z35 | Qinghai | Xunhua | | | 102.49 | 35.85 | 1870 | 812.6 | 9.0 | 54 | 271.8 |
| ZB1 | Z36 | Gansu | Jishishan | | | 103.08 | 35.68 | 1841 | 808.1 | 7.3 | 67 | 501.2 |
| ZB1 | Z42 | Gansu | Gangu | | | 105.34 | 34.73 | 1300 | 874.0 | 10.7 | 68 | 437.3 |
| ZB1 | Z44 | Gansu | Xihe | | | 105.29 | 33.74 | 1086 | 842.4 | 9.1 | 73 | 527.9 |
| ZB1 | Z48 | Sichuan | Hanyuan | | | 102.64 | 29.35 | 939 | 923.9 | 17.8 | 69 | 755.6 |
| ZB1 | Z49 | Sichuan | Mianning | | | 102.19 | 28.29 | 1654 | 820.6 | 14.1 | 70 | 1106.7 |
| ZB1 | Z50 | Sichuan | Yiyuan | | | 101.51 | 27.42 | 2563 | 999.0 | 12.3 | 60 | 828.6 |
| ZB1 | Z51 | Yunnan | Yongping | | | 99.54 | 25.46 | 1667 | 999.0 | 15.8 | 75 | 951.7 |
| ZB1 | Z54 | Guizhou | Zhenfeng | | | 105.63 | 25.61 | 1224 | 894.5 | 16.6 | 79 | 1318.3 |
| ZB1 | Z55 | Guizhou | Qixingguan | | | 105.31 | 27.30 | 1511 | 848.7 | 13.0 | 81 | 866.1 |
| ZB2 | Z30 | Shaanxi | Yangling | | | 108.01 | 34.30 | 528 | 904.8 | 12.1 | 68 | 636.5 |
| ZB2 | Z31 | Shaanxi | Chencang | | | 106.70 | 106.70 | 1050 | 946.1 | 13.5 | 66 | 645.8 |
| ZB2 | Z33 | Shaanxi | Taibai | | | 107.12 | 33.99 | 1401 | 846.5 | 8.1 | 70 | 712.7 |
| ZB2 | Z34 | Shaanxi | Fengxian | | | 106.65 | 33.97 | 1061 | 904.8 | 12.1 | 68 | 636.5 |
| ZB2 | Z37 | Gansu | Yongjing | | | 103.29 | 35.96 | 1633 | 835.3 | 9.7 | 59 | 273.8 |
| ZB2 | Z39 | Gansu | Linxia | | | 103.15 | 35.75 | 1789 | 808.1 | 7.3 | 67 | 501.2 |
| ZB2 | Z40 | Gansu | Jingning | | | 105.73 | 35.52 | 1668 | 834.4 | 7.8 | 66 | 414 |
| ZB2 | Z41 | Gansu | Qin'an | | | 105.80 | 35.06 | 1404 | 880.1 | 10.9 | 66 | 436.3 |
| ZB2 | Z43 | Gansu | Lixian | | | 105.05 | 33.79 | 1473 | 860.3 | 10.3 | 71 | 470.7 |
| ZB2 | Z45 | Gansu | Wudu | | | 110.47 | 35.55 | 452 | 893.6 | 14.9 | 58 | 460.8 |
| ZB2 | Z46 | Sichuan | Songpan | | | 103.60 | 32.66 | 2865 | 720.9 | 6.3 | 63 | 708.3 |
| ZB2 | Z47 | Sichuan | Maoxian | | | 103.85 | 31.68 | 1602 | 840.6 | 11.2 | 74 | 462.3 |
| ZB2 | Z53 | Yunnan | Ludian | | | 103.23 | 27.31 | 1356 | 999.0 | 12.2 | 76 | 852.8 |
| ZA | Z52 | Yunnan | Qiaojiaxian | | | 103.15 | 27.35 | 1733 | 916.2 | 20.8 | 60 | 838.2 |
| ZA | Z56 | Sichuan | Hanyuan | | | 102.39 | 29.68 | 1641 | 923.9 | 17.8 | 69 | 755.6 |
| ZA | Z57 | Sichuan | Wusheng | | | 106.30 | 30.35 | 288 | 977.5 | 17.5 | 84 | 1051.1 |
| ZA | Z58 | Sichuan | Daxian | | | 107.25 | 31.19 | 478 | 974.4 | 17.2 | 80 | 1205.1 |
| ZA | Z59 | Sichuan | Pengxi | | | 105.71 | 30.76 | 366 | 969.4 | 16.8 | 81 | 929.1 |
| ZA | Z60 | Sichuan | Lezhi | | | 104.95 | 30.21 | 424 | 972.7 | 17.3 | 81 | 859.8 |
| ZA | Z61 | Sichuan | Jinyang | | | 103.25 | 27.70 | 1288 | 791.6 | 15.9 | 70 | 795 |
| ZA | Z62 | Chongqing | Jiangjin | | | 106.12 | 29.06 | 250 | 984.0 | 18.3 | 81 | 1000.9 |
| ZA | Z63 | Chongqing | Qianjiang | | | 106.65 | 29.03 | 235 | 945.6 | 15.7 | 79 | 1172.8 |
| ZA | Z64 | Chongqing | Fengdu | | | 107.71 | 30.22 | 397 | 980.3 | 18.3 | 80 | 1018.7 |
| ZA | Z65 | Chongqing | Fengdu | | | 107.71 | 30.22 | 397 | 980.3 | 18.3 | 80 | 1018.7 |
| ZA | Z66 | Yunan | Yongshan | | | 103.63 | 28.23 | 844 | 804.8 | 16.6 | 74 | 684.8 |
| ZA | Z67 | Yunan | Ludian | | | 103.23 | 27.31 | 1356 | 999.0 | 12.2 | 76 | 852.8 |
| ZA | Z68 | Yunan | Qiaojiaxian | | | 102.98 | 27.36 | 1324 | 916.2 | 20.8 | 60 | 838.2 |
| ZA | Z69 | Guizhou | Qixingguan | | | 105.31 | 27.30 | 1511 | 848.7 | 13.0 | 81 | 866.1 |
| ZA | Z70 | Guizhou | Guanling | | | 105.66 | 25.68 | 591 | 887.0 | 16.4 | 79 | 1327.6 |
| ZA | Z71 | Guizhou | Zhenfeng | | | 105.63 | 25.61 | 1224 | 894.5 | 16.6 | 79 | 1318.3 |
| ZA | Z72 | Jiangxi | Xiushui | | | 114.16 | 29.06 | 151 | 998.9 | 16.8 | 79 | 1601.8 |
| Transformed (Z-Score) | |  | |  |  |  |  |  |  |  |  |  |
| Others | Z1 | Shandong | Yiyuan | | | 2.34 | 0.21 | -1.19 | 0.93 | -0.15 | -0.77 | -0.06 |
| Others | Z2 | Shandong | Laicheng | | | 2.19 | 0.23 | -1.22 | 1.06 | 0.09 | -0.77 | -0.04 |
| Others | Z3 | Shandong | Shanting | | | 2.17 | 0.11 | -1.21 | 1.32 | 0.42 | -0.25 | 0.44 |
| Others | Z4 | Hebei | Laishui | | | 1.69 | 0.60 | -1.04 | 1.40 | -0.24 | -0.77 | -0.66 |
| Others | Z5 | Hebei | Tangxian | | | 1.54 | 0.51 | -1.22 | 1.34 | -0.09 | -0.38 | -0.75 |
| Others | Z7 | Hebei | Shexian | | | 1.28 | 0.27 | -0.82 | 0.64 | -0.06 | -0.90 | -0.65 |
| Others | Z8 | Hebei | Fuping | | | 1.41 | 0.51 | -1.08 | 0.96 | -0.12 | -1.55 | -0.51 |
| Others | Z9 | Hebei | Pingshan | | | 1.41 | 0.45 | -1.31 | 0.96 | -0.12 | -1.55 | -0.51 |
| Others | Z10 | Shanxi | Wutai | | | 1.19 | 0.50 | 0.17 | -0.40 | -1.77 | -1.16 | -0.77 |
| Others | Z11 | Shanxi | Xiaoyi | | | 0.86 | 0.33 | -0.29 | 0.13 | -0.59 | -1.42 | -0.98 |
| Others | Z14 | Shanxi | Yangcheng | | | 1.00 | 0.16 | -0.50 | 0.32 | -0.29 | -0.90 | -0.48 |
| Others | Z22 | Shaanxi | Huanglong | | | 0.39 | 0.17 | 0.34 | -0.38 | -1.18 | -0.64 | -0.53 |
| Others | Z38 | Gansu | Dongxiang | | | -1.07 | 0.18 | 1.45 | -2.38 | -2.19 | -0.77 | -0.65 |
| ZB1 | Z6 | Hebei | Shexian | | | 1.28 | 0.27 | -0.82 | 0.64 | -0.06 | -0.90 | -0.65 |
| ZB1 | Z12 | Shanxi | Zaoning | | | 0.61 | 0.19 | -0.16 | -0.18 | -0.74 | -1.16 | -0.72 |
| ZB1 | Z13 | Shanxi | Pingshun | | | 1.28 | 0.25 | -0.58 | -0.38 | -1.06 | -1.16 | -0.58 |
| ZB1 | Z15 | Shanxi | Ruicheng | | | 0.61 | 0.07 | -0.70 | 0.58 | 0.00 | -0.12 | -0.76 |
| ZB1 | Z16 | Henan | Lingbao | | | 0.59 | 0.05 | -0.39 | 0.63 | 0.15 | -0.25 | -0.40 |
| ZB1 | Z17 | Henan | Hubin | | | 0.73 | 0.08 | -0.94 | 0.74 | 0.42 | -0.90 | -0.59 |
| ZB1 | Z18 | Henan | Linzhou | | | 1.35 | 0.24 | -0.88 | 0.92 | 0.06 | -0.25 | -0.21 |
| ZB1 | Z19 | Henan | Baofeng | | | 1.11 | -0.01 | -1.19 | 1.21 | 0.48 | 0.13 | 0.16 |
| ZB1 | Z20 | Shaanxi | Yanchang | | | 0.55 | 0.26 | 0.06 | 0.08 | -0.77 | -0.77 | -0.85 |
| ZB1 | Z21 | Shaanxi | Yichuan | | | 0.54 | 0.20 | -0.23 | 0.03 | -0.80 | -0.90 | -0.72 |
| ZB1 | Z23 | Shaanxi | Hancheng | | | 0.56 | 0.16 | -0.79 | 0.24 | -0.26 | -0.51 | -0.65 |
| ZB1 | Z24 | Shaanxi | Heyang | | | 0.53 | 0.14 | -0.63 | 0.24 | -0.26 | -0.51 | -0.65 |
| ZB1 | Z25 | Shaanxi | Huazhou | | | 0.39 | 0.04 | -0.31 | 0.84 | 0.21 | 0.39 | -0.49 |
| ZB1 | Z26 | Shaanxi | Fuping | | | 0.26 | 0.09 | -0.67 | 0.63 | -0.26 | 0.01 | -0.26 |
| ZB1 | Z27 | Shaanxi | Yaozhou | | | 0.24 | 0.10 | -0.18 | 0.23 | -0.09 | -0.77 | -0.62 |
| ZB1 | Z28 | Shaanxi | Chunhua | | | 0.15 | 0.08 | -0.32 | -0.26 | -0.71 | -0.12 | -0.46 |
| ZB1 | Z29 | Shaanxi | Chunhua | | | 0.15 | 0.08 | -0.32 | -0.26 | -0.71 | -0.12 | -0.46 |
| ZB1 | Z32 | Shaanxi | Chencang | | | -0.34 | 0.06 | -0.02 | 0.40 | 0.15 | -0.25 | -0.23 |
| ZB1 | Z35 | Qinghai | Xunhua | | | -1.24 | 0.19 | 1.55 | -1.59 | -1.18 | -1.80 | -1.65 |
| ZB1 | Z36 | Gansu | Jishishan | | | -1.11 | 0.18 | 1.50 | -1.65 | -1.68 | -0.12 | -0.78 |
| ZB1 | Z42 | Gansu | Gangu | | | -0.60 | 0.08 | 0.61 | -0.67 | -0.68 | 0.01 | -1.02 |
| ZB1 | Z44 | Gansu | Xihe | | | -0.61 | -0.03 | 0.26 | -1.14 | -1.15 | 0.65 | -0.68 |
| ZB1 | Z48 | Sichuan | Hanyuan | | | -1.21 | -0.49 | 0.01 | 0.07 | 1.42 | 0.13 | 0.19 |
| ZB1 | Z49 | Sichuan | Mianning | | | -1.31 | -0.60 | 1.20 | -1.47 | 0.33 | 0.26 | 1.53 |
| ZB1 | Z50 | Sichuan | Yiyuan | | | -1.46 | -0.69 | 2.70 | 1.19 | -0.21 | -1.03 | 0.47 |
| ZB1 | Z51 | Yunnan | Yongping | | | -1.91 | -0.90 | 1.22 | 1.19 | 0.83 | 0.91 | 0.94 |
| ZB1 | Z54 | Guizhou | Zhenfeng | | | -0.53 | -0.88 | 0.49 | -0.37 | 1.07 | 1.43 | 2.34 |
| ZB1 | Z55 | Guizhou | Qixingguan | | | -0.60 | -0.71 | 0.96 | -1.05 | 0.00 | 1.69 | 0.61 |
| ZB2 | Z30 | Shaanxi | Yangling | | | 0.01 | 0.03 | -0.66 | -0.21 | -0.26 | 0.01 | -0.26 |
| ZB2 | Z31 | Shaanxi | Chencang | | | -0.29 | 7.66 | 0.20 | 0.40 | 0.15 | -0.25 | -0.23 |
| ZB2 | Z33 | Shaanxi | Taibai | | | -0.19 | 0.00 | 0.78 | -1.08 | -1.45 | 0.26 | 0.03 |
| ZB2 | Z34 | Shaanxi | Fengxian | | | -0.30 | 0.00 | 0.22 | -0.21 | -0.26 | 0.01 | -0.26 |
| ZB2 | Z37 | Gansu | Yongjing | | | -1.06 | 0.21 | 1.16 | -1.25 | -0.97 | -1.16 | -1.64 |
| ZB2 | Z39 | Gansu | Linxia | | | -1.09 | 0.18 | 1.42 | -1.65 | -1.68 | -0.12 | -0.78 |
| ZB2 | Z40 | Gansu | Jingning | | | -0.51 | 0.16 | 1.22 | -1.26 | -1.54 | -0.25 | -1.11 |
| ZB2 | Z41 | Gansu | Qin'an | | | -0.49 | 0.11 | 0.78 | -0.58 | -0.62 | -0.25 | -1.02 |
| ZB2 | Z43 | Gansu | Lixian | | | -0.66 | -0.02 | 0.90 | -0.88 | -0.80 | 0.39 | -0.89 |
| ZB2 | Z45 | Gansu | Wudu | | | 0.56 | 0.16 | -0.79 | -0.38 | 0.56 | -1.29 | -0.93 |
| ZB2 | Z46 | Sichuan | Songpan | | | -0.99 | -0.14 | 3.19 | -2.95 | -1.98 | -0.64 | 0.01 |
| ZB2 | Z47 | Sichuan | Maoxian | | | -0.93 | -0.25 | 1.11 | -1.17 | -0.53 | 0.78 | -0.93 |
| ZB2 | Z53 | Yunnan | Ludian | | | -1.07 | -0.71 | 0.70 | 1.19 | -0.24 | 1.04 | 0.56 |
| ZA | Z52 | Yunnan | Qiaojiaxian | | | -1.09 | -0.70 | 1.33 | -0.04 | 2.31 | -1.03 | 0.51 |
| ZA | Z56 | Sichuan | Hanyuan | | | -1.26 | -0.46 | 1.17 | 0.07 | 1.42 | 0.13 | 0.19 |
| ZA | Z57 | Sichuan | Wusheng | | | -0.38 | -0.39 | -1.06 | 0.87 | 1.33 | 2.07 | 1.32 |
| ZA | Z58 | Sichuan | Daxian | | | -0.16 | -0.30 | -0.75 | 0.82 | 1.24 | 1.56 | 1.91 |
| ZA | Z59 | Sichuan | Pengxi | | | -0.51 | -0.34 | -0.93 | 0.75 | 1.13 | 1.69 | 0.85 |
| ZA | Z60 | Sichuan | Lezhi | | | -0.68 | -0.40 | -0.84 | 0.80 | 1.27 | 1.69 | 0.59 |
| ZA | Z61 | Sichuan | Jinyang | | | -1.07 | -0.66 | 0.59 | -1.90 | 0.86 | 0.26 | 0.34 |
| ZA | Z62 | Chongqing | Jiangjin | | | -0.42 | -0.52 | -1.12 | 0.97 | 1.57 | 1.69 | 1.13 |
| ZA | Z63 | Chongqing | Qianjiang | | | -0.30 | -0.52 | -1.15 | 0.39 | 0.80 | 1.43 | 1.78 |
| ZA | Z64 | Chongqing | Fengdu | | | -0.06 | -0.40 | -0.88 | 0.91 | 1.57 | 1.56 | 1.20 |
| ZA | Z65 | Chongqing | Fengdu | | | -0.06 | -0.40 | -0.88 | 0.91 | 1.57 | 1.56 | 1.20 |
| ZA | Z66 | Yunan | Yongshan | | | -0.98 | -0.61 | -0.14 | -1.70 | 1.07 | 0.78 | -0.08 |
| ZA | Z67 | Yunan | Ludian | | | -1.07 | -0.71 | 0.70 | 1.19 | -0.24 | 1.04 | 0.56 |
| ZA | Z68 | Yunan | Qiaojiaxian | | | -1.13 | -0.70 | 0.65 | -0.04 | 2.31 | -1.03 | 0.51 |
| ZA | Z69 | Guizhou | Qixingguan | | | -0.60 | -0.71 | 0.96 | -1.05 | 0.00 | 1.69 | 0.61 |
| ZA | Z70 | Guizhou | Guanling | | | -0.52 | -0.88 | -0.56 | -0.48 | 1.01 | 1.43 | 2.37 |
| ZA | Z71 | Guizhou | Zhenfeng | | | -0.53 | -0.88 | 0.49 | -0.37 | 1.07 | 1.43 | 2.34 |
| ZA | Z72 | Jiangxi | Xiushui | | | 1.40 | -0.52 | -1.29 | 1.19 | 1.13 | 1.43 | 3.42 |

ZA is *Z. armatum*; ZB1 samples, belonging to *Z. bungeanum*, are represented by pericarps from Hancheng; ZB2 samples, belonging to *Z. bungeanum*, are represented by pericarps from Fengxian; Others refers to the rest of the samples whose pericarps are red but do not belong to *Z. bungeanum*.

**Table S3** Raw data and transformed (Z-Score) values of soil characteristics in different *Zanthoxylum* plantations

| **Group** | **Code** | **Raw data** | | | | | | | | **Transformed (Z-Score) values** | | | | | | | |
| --- | --- | --- | --- | --- | --- | --- | --- | --- | --- | --- | --- | --- | --- | --- | --- | --- | --- |
|  |  | **pH** | **OM** | **N_t_** | **P_t_** | **K_t_** | **N_a_** | **P_a_** | **K_a_** | **pH** | **OM** | **N_t_** | **P_t_** | **K_t_** | **N_a_** | **P_a_** | **K_a_** |
| Others | Z1 | 7.80 | 44.46 | 2.21 | 0.62 | 16.22 | 79.38 | 4.28 | 320.30 | 0.02 | 0.52 | 0.72 | -0.72 | 0.49 | 0.06 | -0.67 | -0.30 |
| Others | Z2 | 7.47 | 27.89 | 1.35 | 1.65 | 16.62 | 48.58 | 175.13 | 161.30 | -0.34 | -0.20 | -0.18 | 0.68 | 0.60 | -0.33 | 2.06 | -1.01 |
| Others | Z3 | 7.14 | 41.70 | 1.85 | 0.77 | 13.44 | 80.78 | 28.28 | 253.50 | -0.70 | 0.40 | 0.35 | -0.51 | -0.22 | 0.07 | -0.29 | -0.60 |
| Others | Z4 | 8.05 | 58.14 | 2.39 | 1.08 | 14.05 | 80.78 | 54.63 | 580.50 | 0.29 | 1.12 | 0.89 | -0.10 | -0.07 | 0.07 | 0.13 | 0.87 |
| Others | Z5 | 8.13 | 48.45 | 1.94 | 1.25 | 12.72 | 84.98 | 55.88 | 822.00 | 0.38 | 0.70 | 0.44 | 0.13 | -0.41 | 0.13 | 0.15 | 1.96 |
| Others | Z7 | 8.28 | 26.88 | 1.09 | 0.58 | 11.97 | 51.38 | 2.38 | 277.80 | 0.54 | -0.25 | -0.44 | -0.78 | -0.60 | -0.30 | -0.70 | -0.49 |
| Others | Z8 | 8.52 | 17.01 | 0.80 | 1.24 | 11.15 | 54.18 | 55.63 | 749.70 | 0.80 | -0.68 | -0.74 | 0.12 | -0.81 | -0.26 | 0.15 | 1.63 |
| Others | Z9 | 7.98 | 25.98 | 1.39 | 0.77 | 14.38 | 75.18 | 32.88 | 328.80 | 0.21 | -0.29 | -0.13 | -0.52 | 0.02 | 0.00 | -0.21 | -0.26 |
| Others | Z10 | 7.77 | 47.78 | 2.42 | 1.07 | 14.44 | 87.78 | 15.98 | 351.80 | -0.01 | 0.67 | 0.93 | -0.11 | 0.04 | 0.16 | -0.48 | -0.16 |
| Others | Z11 | 8.41 | 20.56 | 0.81 | 0.66 | 11.29 | 30.38 | 2.18 | 260.80 | 0.68 | -0.52 | -0.73 | -0.67 | -0.78 | -0.56 | -0.70 | -0.56 |
| Others | Z14 | 7.80 | 111.70 | 4.51 | 0.84 | 11.82 | 180.18 | 31.13 | 652.20 | 0.02 | 3.48 | 3.08 | -0.43 | -0.64 | 1.33 | -0.24 | 1.19 |
| Others | Z22 | 8.18 | 31.85 | 1.50 | 0.71 | 9.69 | 59.78 | 11.68 | 310.60 | 0.43 | -0.03 | -0.01 | -0.60 | -1.19 | -0.19 | -0.55 | -0.34 |
| Others | Z38 | 8.51 | 14.85 | 0.70 | 0.74 | 14.74 | 33.18 | 5.83 | 197.70 | 0.79 | -0.77 | -0.84 | -0.55 | 0.11 | -0.53 | -0.64 | -0.85 |
| ZB1 | Z6 | 8.24 | 35.54 | 1.67 | 0.66 | 13.99 | 58.38 | 1.83 | 288.70 | 0.50 | 0.13 | 0.15 | -0.67 | -0.08 | -0.21 | -0.71 | -0.44 |
| ZB1 | Z12 | 8.10 | 18.17 | 0.89 | 0.80 | 10.47 | 40.18 | 28.78 | 236.50 | 0.35 | -0.63 | -0.65 | -0.48 | -0.99 | -0.44 | -0.28 | -0.67 |
| ZB1 | Z13 | 8.57 | 35.12 | 1.58 | 1.70 | 19.87 | 68.18 | 5.43 | 230.50 | 0.86 | 0.11 | 0.07 | 0.75 | 1.43 | -0.09 | -0.65 | -0.70 |
| ZB1 | Z15 | 8.36 | 13.42 | 0.66 | 0.79 | 12.69 | 9.38 | 2.13 | 632.70 | 0.63 | -0.84 | -0.88 | -0.49 | -0.42 | -0.83 | -0.70 | 1.11 |
| ZB1 | Z16 | 8.28 | 15.69 | 0.80 | 0.89 | 9.82 | 38.78 | 8.33 | 374.90 | 0.54 | -0.74 | -0.74 | -0.36 | -1.16 | -0.46 | -0.60 | -0.05 |
| ZB1 | Z17 | 8.46 | 15.80 | 0.73 | 0.58 | 12.10 | 27.58 | 2.93 | 323.90 | 0.74 | -0.73 | -0.81 | -0.78 | -0.57 | -0.60 | -0.69 | -0.28 |
| ZB1 | Z18 | 8.09 | 20.49 | 0.63 | 0.47 | 12.90 | 24.78 | 8.83 | 349.40 | 0.33 | -0.53 | -0.91 | -0.93 | -0.36 | -0.63 | -0.60 | -0.17 |
| ZB1 | Z19 | 8.42 | 10.82 | 0.46 | 0.77 | 11.79 | 14.98 | 4.83 | 195.30 | 0.69 | -0.95 | -1.09 | -0.52 | -0.65 | -0.76 | -0.66 | -0.86 |
| ZB1 | Z20 | 8.40 | 11.32 | 0.64 | 0.67 | 11.08 | 31.78 | 7.93 | 269.30 | 0.67 | -0.93 | -0.90 | -0.66 | -0.83 | -0.54 | -0.61 | -0.53 |
| ZB1 | Z21 | 8.67 | 11.42 | 0.50 | 0.51 | 11.50 | 19.18 | 1.48 | 201.40 | 0.97 | -0.92 | -1.05 | -0.87 | -0.72 | -0.70 | -0.71 | -0.83 |
| ZB1 | Z23 | 8.25 | 43.41 | 1.25 | 0.85 | 10.63 | 41.58 | 17.93 | 280.20 | 0.51 | 0.48 | -0.27 | -0.41 | -0.95 | -0.42 | -0.45 | -0.48 |
| ZB1 | Z24 | 8.52 | 18.99 | 0.88 | 1.06 | 11.05 | 37.38 | 57.53 | 567.30 | 0.80 | -0.59 | -0.66 | -0.12 | -0.84 | -0.47 | 0.18 | 0.81 |
| ZB1 | Z25 | 8.39 | 31.20 | 1.06 | 0.82 | 10.84 | 39.48 | 37.73 | 423.75 | 0.66 | -0.06 | -0.47 | -0.45 | -0.89 | -0.45 | -0.14 | 0.17 |
| ZB1 | Z26 | 7.87 | 19.89 | 1.12 | 0.76 | 10.78 | 75.18 | 97.63 | 573.30 | 0.10 | -0.55 | -0.41 | -0.54 | -0.91 | 0.00 | 0.82 | 0.84 |
| ZB1 | Z27 | 8.50 | 20.08 | 0.96 | 0.80 | 11.13 | 42.98 | 7.48 | 123.70 | 0.78 | -0.55 | -0.57 | -0.48 | -0.82 | -0.40 | -0.62 | -1.18 |
| ZB1 | Z28 | 8.43 | 18.66 | 0.89 | 0.79 | 10.71 | 40.18 | 8.88 | 167.40 | 0.71 | -0.61 | -0.65 | -0.50 | -0.93 | -0.44 | -0.59 | -0.98 |
| ZB1 | Z29 | 8.31 | 18.58 | 0.92 | 0.82 | 11.55 | 45.78 | 12.83 | 189.20 | 0.57 | -0.61 | -0.61 | -0.45 | -0.71 | -0.37 | -0.53 | -0.89 |
| ZB1 | Z32 | 8.20 | 16.35 | 0.86 | 0.86 | 14.63 | 33.18 | 13.08 | 327.50 | 0.45 | -0.71 | -0.67 | -0.40 | 0.08 | -0.53 | -0.53 | -0.27 |
| ZB1 | Z35 | 8.27 | 20.54 | 1.03 | 0.86 | 11.80 | 35.98 | 11.05 | 220.75 | 0.53 | -0.53 | -0.50 | -0.40 | -0.64 | -0.49 | -0.56 | -0.74 |
| ZB1 | Z36 | 8.63 | 14.53 | 0.79 | 0.87 | 12.16 | 23.38 | 16.58 | 310.60 | 0.92 | -0.79 | -0.75 | -0.38 | -0.55 | -0.65 | -0.47 | -0.34 |
| ZB1 | Z42 | 8.28 | 19.52 | 1.23 | 1.96 | 15.92 | 51.38 | 67.13 | 698.70 | 0.54 | -0.57 | -0.30 | 1.10 | 0.42 | -0.30 | 0.33 | 1.40 |
| ZB1 | Z44 | 8.51 | 10.37 | 0.56 | 0.84 | 15.70 | 30.38 | 46.68 | 175.90 | 0.79 | -0.97 | -0.99 | -0.42 | 0.36 | -0.56 | 0.01 | -0.95 |
| ZB1 | Z48 | 5.16 | 60.46 | 2.80 | 3.00 | 7.88 | 128.38 | 305.88 | 684.30 | -2.86 | 1.23 | 1.31 | 2.51 | -1.66 | 0.67 | 4.14 | 1.34 |
| ZB1 | Z49 | 6.29 | 61.02 | 2.89 | 3.27 | 18.69 | 145.18 | 295.88 | 558.60 | -1.63 | 1.25 | 1.41 | 2.88 | 1.13 | 0.88 | 3.98 | 0.77 |
| ZB1 | Z50 | 6.03 | 51.77 | 2.25 | 1.77 | 8.61 | 89.18 | 77.08 | 546.00 | -1.91 | 0.85 | 0.76 | 0.84 | -1.47 | 0.18 | 0.49 | 0.72 |
| ZB1 | Z51 | 5.63 | 109.88 | 4.30 | 0.90 | 13.50 | 208.18 | 21.93 | 327.50 | -2.34 | 3.39 | 2.86 | -0.34 | -0.21 | 1.68 | -0.39 | -0.27 |
| ZB1 | Z54 | 5.83 | 57.75 | 2.65 | 2.84 | 11.73 | 120.91 | 226.28 | 596.30 | -2.13 | 1.11 | 1.16 | 2.30 | -0.66 | 0.58 | 2.87 | 0.94 |
| ZB1 | Z55 | 8.30 | 19.19 | 1.04 | 1.85 | 16.02 | 46.13 | 60.31 | 332.08 | 0.57 | -0.58 | -0.49 | 0.95 | 0.44 | -0.36 | 0.23 | -0.24 |
| ZB2 | Z30 | 8.27 | 21.03 | 1.22 | 0.96 | 11.59 | 49.98 | 12.18 | 587.70 | 0.53 | -0.50 | -0.31 | -0.26 | -0.70 | -0.31 | -0.54 | 0.90 |
| ZB2 | Z31 | 8.14 | 22.57 | 1.21 | 1.24 | 13.80 | 48.58 | 64.98 | 281.40 | 0.39 | -0.44 | -0.32 | 0.12 | -0.13 | -0.33 | 0.30 | -0.47 |
| ZB2 | Z33 | 8.13 | 23.13 | 1.25 | 0.79 | 11.73 | 19.18 | 15.08 | 200.10 | 0.38 | -0.41 | -0.28 | -0.49 | -0.66 | -0.70 | -0.50 | -0.84 |
| ZB2 | Z34 | 8.40 | 17.94 | 0.81 | 0.95 | 11.88 | 52.78 | 7.03 | 241.40 | 0.67 | -0.64 | -0.72 | -0.27 | -0.63 | -0.28 | -0.62 | -0.65 |
| ZB2 | Z37 | 8.75 | 9.17 | 0.38 | 0.73 | 13.98 | 12.18 | 5.08 | 211.10 | 1.05 | -1.02 | -1.17 | -0.57 | -0.08 | -0.79 | -0.66 | -0.79 |
| ZB2 | Z39 | 8.35 | 10.40 | 0.49 | 0.83 | 13.34 | 608.58 | 10.08 | 256.00 | 0.62 | -0.97 | -1.06 | -0.43 | -0.25 | 6.72 | -0.58 | -0.59 |
| ZB2 | Z40 | 8.26 | 64.61 | 3.27 | 1.05 | 14.26 | 115.78 | 10.73 | 618.60 | 0.52 | 1.41 | 1.80 | -0.14 | -0.01 | 0.51 | -0.57 | 1.04 |
| ZB2 | Z41 | 8.44 | 12.33 | 0.60 | 1.00 | 15.39 | 24.78 | 46.38 | 807.90 | 0.72 | -0.89 | -0.94 | -0.20 | 0.28 | -0.63 | 0.00 | 1.89 |
| ZB2 | Z43 | 8.21 | 19.58 | 0.94 | 1.96 | 16.61 | 35.98 | 83.63 | 349.40 | 0.47 | -0.57 | -0.59 | 1.10 | 0.59 | -0.49 | 0.60 | -0.17 |
| ZB2 | Z45 | 8.21 | 27.31 | 1.43 | 1.01 | 15.85 | 66.78 | 43.83 | 104.30 | 0.47 | -0.23 | -0.08 | -0.20 | 0.40 | -0.10 | -0.04 | -1.27 |
| ZB2 | Z46 | 8.02 | 5.83 | 0.44 | 0.71 | 15.04 | 13.58 | 0.58 | 41.20 | 0.26 | -1.17 | -1.11 | -0.60 | 0.19 | -0.77 | -0.73 | -1.55 |
| ZB2 | Z47 | 7.31 | 37.17 | 2.01 | 1.28 | 14.86 | 98.98 | 99.38 | 343.30 | -0.51 | 0.20 | 0.51 | 0.18 | 0.14 | 0.30 | 0.85 | -0.19 |
| ZB2 | Z53 | 7.26 | 31.57 | 1.41 | 0.90 | 26.11 | 117.18 | 61.03 | 570.90 | -0.57 | -0.04 | -0.11 | -0.35 | 3.04 | 0.53 | 0.24 | 0.83 |
| ZA | Z52 | 7.16 | 32.05 | 1.62 | 0.43 | 17.23 | 77.98 | 1.23 | 299.60 | -0.68 | -0.02 | 0.11 | -0.98 | 0.76 | 0.04 | -0.72 | -0.39 |
| ZA | Z56 | 7.22 | 104.63 | 4.95 | 5.04 | 15.62 | 262.78 | 190.38 | 982.50 | -0.61 | 3.16 | 3.53 | 5.29 | 0.34 | 2.37 | 2.30 | 2.68 |
| ZA | Z57 | 5.63 | 21.06 | 1.42 | 1.15 | 10.72 | 192.78 | 94.88 | 1095.60 | -2.34 | -0.50 | -0.10 | 0.00 | -0.93 | 1.48 | 0.78 | 3.19 |
| ZA | Z58 | 6.80 | 22.40 | 1.09 | 0.52 | 16.16 | 77.98 | 3.18 | 101.90 | -1.07 | -0.44 | -0.44 | -0.86 | 0.48 | 0.04 | -0.69 | -1.28 |
| ZA | Z59 | 8.09 | 24.31 | 1.32 | 1.29 | 17.10 | 52.78 | 32.88 | 642.30 | 0.33 | -0.36 | -0.21 | 0.18 | 0.72 | -0.28 | -0.21 | 1.15 |
| ZA | Z60 | 7.46 | 18.25 | 1.11 | 1.25 | 17.23 | 48.58 | 13.48 | 243.80 | -0.35 | -0.63 | -0.41 | 0.14 | 0.75 | -0.33 | -0.52 | -0.64 |
| ZA | Z61 | 4.02 | 52.93 | 2.35 | 0.93 | 15.88 | 104.58 | 134.13 | 334.80 | -4.10 | 0.90 | 0.86 | -0.30 | 0.41 | 0.37 | 1.40 | -0.23 |
| ZA | Z62 | 7.93 | 24.47 | 1.31 | 2.71 | 18.13 | 56.98 | 61.48 | 283.90 | 0.16 | -0.35 | -0.21 | 2.12 | 0.99 | -0.23 | 0.24 | -0.46 |
| ZA | Z63 | 7.79 | 12.76 | 0.48 | 0.79 | 21.05 | 44.38 | 20.13 | 155.30 | 0.01 | -0.87 | -1.07 | -0.50 | 1.74 | -0.39 | -0.42 | -1.04 |
| ZA | Z64 | 7.86 | 18.62 | 0.89 | 1.22 | 19.59 | 50.68 | 40.80 | 219.60 | 0.08 | -0.61 | -0.64 | 0.09 | 1.36 | -0.31 | -0.09 | -0.75 |
| ZA | Z65 | 7.95 | 62.90 | 3.05 | 1.56 | 24.08 | 91.98 | 31.38 | 533.10 | 0.18 | 1.33 | 1.57 | 0.55 | 2.52 | 0.21 | -0.24 | 0.66 |
| ZA | Z66 | 7.91 | 27.79 | 1.40 | 1.06 | 25.51 | 44.38 | 19.58 | 239.00 | 0.14 | -0.21 | -0.12 | -0.12 | 2.89 | -0.39 | -0.42 | -0.66 |
| ZA | Z67 | 7.93 | 45.34 | 2.22 | 1.21 | 24.80 | 68.18 | 25.48 | 386.05 | 0.16 | 0.56 | 0.72 | 0.08 | 2.70 | -0.09 | -0.33 | 0.00 |
| ZA | Z68 | 7.77 | 72.82 | 3.20 | 1.44 | 13.98 | 140.98 | 90.38 | 746.10 | -0.01 | 1.77 | 1.73 | 0.40 | -0.08 | 0.83 | 0.70 | 1.62 |
| ZA | Z69 | 7.41 | 64.64 | 2.48 | 0.99 | 10.46 | 59.78 | 4.28 | 92.20 | -0.41 | 1.41 | 0.99 | -0.22 | -0.99 | -0.19 | -0.67 | -1.32 |
| ZA | Z70 | 7.75 | 32.10 | 2.14 | 1.24 | 12.76 | 69.58 | 83.63 | 630.30 | -0.04 | -0.02 | 0.64 | 0.11 | -0.40 | -0.07 | 0.60 | 1.09 |
| ZA | Z71 | 6.85 | 42.31 | 2.06 | 0.81 | 21.23 | 105.98 | 4.33 | 550.50 | -1.02 | 0.43 | 0.56 | -0.46 | 1.79 | 0.39 | -0.67 | 0.74 |
| ZA | Z72 | 6.06 | 40.94 | 1.69 | 1.13 | 11.88 | 68.18 | 84.38 | 209.90 | -1.88 | 0.37 | 0.18 | -0.03 | -0.62 | -0.09 | 0.61 | -0.79 |

ZA is *Z. armatum*; ZB1 samples, belonging to *Z. bungeanum*, are represented by pericarps from Hancheng; ZB2 samples, belonging to *Z. bungeanum*, are represented by pericarps from Fengxian; Others refers to the rest of the samples whose pericarps are red but do not belong to *Z. bungeanum*.

**Table S4** Raw data and transformed (Z-Score) values of fatty acid date in different *Zanthoxylum* pericarps

| **Group** | **Code** | **C16:0** | **C18:0** | **C20:0** | **C22:0** | **C16:1** | **C18:1n9** | **C18:1n13t** | **C20:1** | **C18:2** |
| --- | --- | --- | --- | --- | --- | --- | --- | --- | --- | --- |
| Others | Z1 | 22.93 | 9.77 | 1.96 | 4.24 | 10.2 | 19.11 | 14.12 | 1.46 | 21.98 |
| Others | Z2 | 35.82 | 19.86 | 2.18 | 4.63 | 20.35 | 47.4 | 28.41 | 3.19 | 50.93 |
| Others | Z3 | 33.1 | 16.03 | 2.08 | 5.58 | 22.8 | 38.85 | 28.38 | 1.94 | 42.95 |
| Others | Z4 | 21.33 | 10.47 | 1.39 | 7.14 | 4.24 | 18.26 | 6.99 | 1.5 | 26.61 |
| Others | Z5 | 25.18 | 9.43 | 1.19 | 4.3 | 7.34 | 25.63 | 10.33 | 1.23 | 36.58 |
| Others | Z7 | 24.74 | 11.32 | 1.34 | 4.4 | 12.27 | 25.46 | 12.02 | 1.9 | 30.27 |
| Others | Z8 | 22.03 | 9.89 | 1.09 | 4.55 | 7.02 | 20.98 | 7.8 | 1.43 | 25.81 |
| Others | Z9 | 73.13 | 39.52 | 2.97 | 5.71 | 55.04 | 108.94 | 72.2 | 7.31 | 113.01 |
| Others | Z10 | 55.38 | 52.13 | 4.5 | 8.81 | 17.76 | 86.59 | 38.78 | 4.75 | 148.2 |
| Others | Z11 | 42.91 | 17.95 | 1.58 | 4.78 | 39.02 | 56.37 | 36.21 | 1.46 | 51.43 |
| Others | Z14 | 32.11 | 15.39 | 0.27 | 5.15 | 11.75 | 35.21 | 16.2 | 2.5 | 45.27 |
| Others | Z22 | 28.04 | 9.49 | 1.33 | 4.06 | 17.4 | 27.29 | 20.04 | 1.75 | 0 |
| Others | Z38 | 41.95 | 16.06 | 1.59 | 4.95 | 6.86 | 52.69 | 12.42 | 2.53 | 45.03 |
| ZB1 | Z6 | 36.73 | 16.37 | 1.37 | 4.9 | 29.86 | 50.71 | 33.08 | 1.41 | 37.93 |
| ZB1 | Z12 | 43.44 | 16.81 | 1.64 | 4.65 | 38.37 | 50.24 | 47.16 | 2.8 | 53.08 |
| ZB1 | Z13 | 0 | 3.26 | 0.29 | 2.03 | 0 | 26.56 | 29.91 | 2.28 | 0 |
| ZB1 | Z15 | 80.26 | 41.2 | 5.77 | 6.47 | 73.47 | 99.03 | 72.36 | 5.88 | 108.77 |
| ZB1 | Z16 | 34.86 | 13.09 | 2.48 | 5.25 | 21.37 | 41.8 | 28.96 | 1.85 | 32.64 |
| ZB1 | Z17 | 37.38 | 16.08 | 1.68 | 4.94 | 30.58 | 45.32 | 32.33 | 2.61 | 52.06 |
| ZB1 | Z18 | 39.45 | 14.59 | 1.42 | 4.66 | 41.83 | 50.85 | 45.99 | 3.24 | 56.79 |
| ZB1 | Z19 | 44.8 | 15.34 | 1.4 | 4.11 | 44 | 56.65 | 52.29 | 2.77 | 45.01 |
| ZB1 | Z20 | 27.61 | 11.33 | 1.59 | 5 | 11.2 | 29.25 | 19.79 | 1.33 | 38.73 |
| ZB1 | Z21 | 24.6 | 8.87 | 0.27 | 4.26 | 12.9 | 36.04 | 22.6 | 1.35 | 29.74 |
| ZB1 | Z23 | 33.58 | 12.44 | 1.37 | 4.82 | 20.62 | 25.28 | 28.96 | 1.4 | 27.17 |
| ZB1 | Z24 | 34.31 | 13.34 | 1.14 | 4.19 | 21.26 | 48.22 | 29.79 | 1.3 | 41.79 |
| ZB1 | Z25 | 33.8 | 14.66 | 1.25 | 4.65 | 23.44 | 43.87 | 32.28 | 2.43 | 36.95 |
| ZB1 | Z26 | 39.29 | 14.08 | 1.63 | 5.25 | 36.06 | 37.78 | 36.75 | 1.34 | 40.73 |
| ZB1 | Z27 | 35 | 12.93 | 1.51 | 5.42 | 28.1 | 34.71 | 28.28 | 1.29 | 31.25 |
| ZB1 | Z28 | 22.58 | 2.56 | 1.18 | 1.68 | 10.25 | 23.72 | 18.99 | 1.2 | 0 |
| ZB1 | Z29 | 24.44 | 9.84 | 1.57 | 1.79 | 10.37 | 29.59 | 20.73 | 1.28 | 22.23 |
| ZB1 | Z32 | 44.72 | 16.2 | 1.94 | 5.56 | 41.97 | 55.79 | 41.66 | 1.42 | 46.98 |
| ZB1 | Z35 | 34.79 | 12.22 | 1.85 | 4.03 | 32.16 | 39.86 | 31.79 | 1.27 | 37.49 |
| ZB1 | Z36 | 29.13 | 11.46 | 1.41 | 4.31 | 5.53 | 33.47 | 7.81 | 1.32 | 23.36 |
| ZB1 | Z42 | 125.03 | 50.24 | 3.19 | 4.04 | 139.13 | 138.65 | 121.07 | 5.55 | 166.06 |
| ZB1 | Z44 | 29.52 | 11 | 0.26 | 1.83 | 7.56 | 36.31 | 8.23 | 1.3 | 28.51 |
| ZB1 | Z48 | 60.95 | 20.24 | 1.76 | 5.44 | 56.07 | 52.4 | 32.11 | 2.22 | 59.27 |
| ZB1 | Z49 | 32.44 | 12.82 | 1.77 | 5.63 | 16.73 | 19.84 | 13.74 | 1.35 | 32.83 |
| ZB1 | Z50 | 67.37 | 26.13 | 1.86 | 3.82 | 68.07 | 74.11 | 173.36 | 1.35 | 66.69 |
| ZB1 | Z51 | 32.4 | 13.55 | 1.85 | 5.23 | 15.48 | 22.34 | 9.49 | 1.45 | 32.07 |
| ZB1 | Z54 | 105.4 | 107.21 | 9.28 | 15.55 | 57.11 | 181.37 | 83.28 | 7.44 | 305.32 |
| ZB1 | Z55 | 57.12 | 48.55 | 4.55 | 7.57 | 28.45 | 88.88 | 46.82 | 4.01 | 142.19 |
| ZB2 | Z30 | 38.2 | 14.09 | 1.44 | 5.23 | 8.6 | 44.21 | 8.97 | 1.24 | 37.35 |
| ZB2 | Z31 | 26.71 | 10.54 | 0.25 | 4.88 | 6.2 | 23.45 | 9.06 | 1.25 | 24.77 |
| ZB2 | Z33 | 30.07 | 11.1 | 1.96 | 5.15 | 6.7 | 30.86 | 8.76 | 1.27 | 27.64 |
| ZB2 | Z34 | 34.31 | 13.56 | 1.85 | 5.35 | 5.95 | 30.88 | 10.43 | 1.27 | 28.66 |
| ZB2 | Z37 | 31.72 | 11.72 | 1.5 | 5.19 | 9.06 | 31.66 | 11.88 | 1.33 | 30 |
| ZB2 | Z39 | 33.46 | 13.72 | 1.39 | 4.63 | 5.68 | 33.79 | 12.04 | 1.23 | 29.46 |
| ZB2 | Z40 | 34.12 | 15.68 | 1.19 | 4.17 | 6.6 | 42.8 | 10.24 | 1.31 | 40.79 |
| ZB2 | Z41 | 41.31 | 18.61 | 0.62 | 5.29 | 8.34 | 53 | 12.66 | 1.36 | 46.15 |
| ZB2 | Z43 | 36.53 | 13.96 | 1.72 | 4.75 | 10.75 | 45.2 | 11.08 | 1.33 | 35.12 |
| ZB2 | Z45 | 42.76 | 14.64 | 1.97 | 5.2 | 8.15 | 48.69 | 14.97 | 2.35 | 43.25 |
| ZB2 | Z46 | 26.2 | 11.97 | 1.4 | 4.7 | 5.51 | 23.44 | 6.22 | 1.48 | 24.61 |
| ZB2 | Z47 | 34.59 | 13 | 1.43 | 4.31 | 5.92 | 34.55 | 9.46 | 1.46 | 27.24 |
| ZB2 | Z53 | 37.46 | 13.88 | 1.4 | 4.87 | 27.81 | 27.59 | 21.18 | 1.44 | 32.08 |
| ZA | Z52 | 30.19 | 11.95 | 1.88 | 4.79 | 40.5 | 13.57 | 22.73 | 1.36 | 42.06 |
| ZA | Z56 | 52.87 | 19.74 | 0.28 | 1.98 | 148.33 | 32.86 | 92.73 | 1.41 | 52.51 |
| ZA | Z57 | 29.18 | 11.62 | 1.4 | 3.69 | 18.75 | 10.83 | 12.14 | 1.48 | 31.61 |
| ZA | Z58 | 31.33 | 11.33 | 0.28 | 3.27 | 94.83 | 14.01 | 50.15 | 1.42 | 27.85 |
| ZA | Z59 | 26.33 | 11.32 | 1.18 | 3.31 | 54.62 | 12.05 | 30.57 | 1.43 | 24.45 |
| ZA | Z60 | 29.49 | 12.93 | 1.41 | 3.7 | 51.72 | 13.19 | 29.1 | 1.44 | 26.88 |
| ZA | Z61 | 27.67 | 13.05 | 1.12 | 3.5 | 31.7 | 12.79 | 17.08 | 1.46 | 25.47 |
| ZA | Z62 | 22.65 | 9.62 | 1.12 | 3.67 | 32.53 | 14.96 | 16.15 | 1.39 | 22.64 |
| ZA | Z63 | 26.64 | 11.35 | 1.22 | 2.1 | 26.29 | 10.66 | 16.65 | 1.5 | 29.91 |
| ZA | Z64 | 32.84 | 16.29 | 1.94 | 4.55 | 42.44 | 17.65 | 24.34 | 1.41 | 29.93 |
| ZA | Z65 | 53.27 | 17.97 | 1.59 | 4.18 | 159.87 | 25.62 | 87.16 | 1.47 | 39.13 |
| ZA | Z66 | 57.67 | 18.59 | 0.29 | 5.02 | 143.01 | 24.85 | 75.25 | 1.47 | 43.85 |
| ZA | Z67 | 69.81 | 28.28 | 1.99 | 4.15 | 232.46 | 44.23 | 136.08 | 1.45 | 66.47 |
| ZA | Z68 | 64.18 | 28.64 | 3.25 | 5.41 | 201.69 | 44.92 | 109.24 | 2.36 | 76.12 |
| ZA | Z69 | 55.73 | 29.17 | 0.29 | 3.49 | 178.02 | 42.83 | 96.88 | 1.45 | 69.74 |
| ZA | Z70 | 41.71 | 20.48 | 2.94 | 5.32 | 86.29 | 21.87 | 42.04 | 1.48 | 41.63 |
| ZA | Z71 | 33.36 | 12.69 | 0.28 | 4.02 | 76.59 | 15.06 | 41.9 | 1.38 | 33.68 |
| ZA | Z72 | 33.24 | 11.5 | 1.37 | 3.48 | 18.08 | 20.6 | 16.51 | 1.49 | 24.67 |
| Transformed (Z-Score) | | |  |  |  |  |  |  |  |  |
| Others | Z1 | -0.87 | -0.56 | 0.19 | -0.26 | -0.61 | -0.74 | -0.63 | -0.4 | -0.58 |
| Others | Z2 | -0.19 | 0.15 | 0.36 | -0.04 | -0.41 | 0.25 | -0.2 | 0.92 | 0.1 |
| Others | Z3 | -0.33 | -0.12 | 0.28 | 0.49 | -0.36 | -0.05 | -0.2 | -0.03 | -0.09 |
| Others | Z4 | -0.96 | -0.51 | -0.24 | 1.37 | -0.74 | -0.77 | -0.85 | -0.37 | -0.47 |
| Others | Z5 | -0.75 | -0.58 | -0.39 | -0.23 | -0.67 | -0.51 | -0.75 | -0.57 | -0.24 |
| Others | Z7 | -0.78 | -0.45 | -0.27 | -0.17 | -0.57 | -0.52 | -0.69 | -0.06 | -0.39 |
| Others | Z8 | -0.92 | -0.55 | -0.46 | -0.09 | -0.68 | -0.67 | -0.82 | -0.42 | -0.49 |
| Others | Z9 | 1.79 | 1.52 | 0.95 | 0.57 | 0.3 | 2.39 | 1.13 | 4.06 | 1.55 |
| Others | Z10 | 0.85 | 2.4 | 2.1 | 2.31 | -0.46 | 1.61 | 0.12 | 2.11 | 2.37 |
| Others | Z11 | 0.19 | 0.02 | -0.09 | 0.04 | -0.03 | 0.56 | 0.04 | -0.4 | 0.11 |
| Others | Z14 | -0.39 | -0.16 | -1.08 | 0.25 | -0.58 | -0.18 | -0.57 | 0.4 | -0.04 |
| Others | Z22 | -0.6 | -0.57 | -0.28 | -0.36 | -0.47 | -0.45 | -0.45 | -0.18 | -1.09 |
| Others | Z38 | 0.14 | -0.12 | -0.09 | 0.14 | -0.68 | 0.43 | -0.68 | 0.42 | -0.04 |
| ZB1 | Z6 | -0.14 | -0.09 | -0.25 | 0.11 | -0.21 | 0.36 | -0.06 | -0.43 | -0.21 |
| ZB1 | Z12 | 0.22 | -0.06 | -0.05 | -0.03 | -0.04 | 0.35 | 0.37 | 0.62 | 0.15 |
| ZB1 | Z13 | -2.09 | -1.01 | -1.06 | -1.51 | -0.82 | -0.48 | -0.15 | 0.23 | -1.09 |
| ZB1 | Z15 | 2.17 | 1.64 | 3.06 | 0.99 | 0.67 | 2.05 | 1.13 | 2.97 | 1.45 |
| ZB1 | Z16 | -0.24 | -0.32 | 0.58 | 0.31 | -0.39 | 0.05 | -0.18 | -0.1 | -0.33 |
| ZB1 | Z17 | -0.11 | -0.12 | -0.02 | 0.13 | -0.2 | 0.18 | -0.08 | 0.48 | 0.12 |
| ZB1 | Z18 | 0 | -0.22 | -0.21 | -0.03 | 0.03 | 0.37 | 0.33 | 0.96 | 0.23 |
| ZB1 | Z19 | 0.29 | -0.17 | -0.23 | -0.33 | 0.07 | 0.57 | 0.52 | 0.6 | -0.04 |
| ZB1 | Z20 | -0.62 | -0.45 | -0.09 | 0.17 | -0.59 | -0.38 | -0.46 | -0.49 | -0.19 |
| ZB1 | Z21 | -0.78 | -0.62 | -1.08 | -0.25 | -0.56 | -0.15 | -0.37 | -0.48 | -0.4 |
| ZB1 | Z23 | -0.31 | -0.37 | -0.25 | 0.06 | -0.4 | -0.52 | -0.18 | -0.44 | -0.46 |
| ZB1 | Z24 | -0.27 | -0.31 | -0.42 | -0.29 | -0.39 | 0.28 | -0.16 | -0.52 | -0.12 |
| ZB1 | Z25 | -0.3 | -0.21 | -0.34 | -0.03 | -0.34 | 0.13 | -0.08 | 0.34 | -0.23 |
| ZB1 | Z26 | 0 | -0.25 | -0.06 | 0.31 | -0.09 | -0.09 | 0.05 | -0.49 | -0.14 |
| ZB1 | Z27 | -0.23 | -0.33 | -0.15 | 0.4 | -0.25 | -0.19 | -0.2 | -0.53 | -0.36 |
| ZB1 | Z28 | -0.89 | -1.06 | -0.39 | -1.7 | -0.61 | -0.58 | -0.48 | -0.59 | -1.09 |
| ZB1 | Z29 | -0.79 | -0.55 | -0.1 | -1.64 | -0.61 | -0.37 | -0.43 | -0.53 | -0.57 |
| ZB1 | Z32 | 0.28 | -0.11 | 0.18 | 0.48 | 0.03 | 0.54 | 0.2 | -0.43 | 0 |
| ZB1 | Z35 | -0.24 | -0.38 | 0.11 | -0.38 | -0.17 | -0.01 | -0.1 | -0.54 | -0.22 |
| ZB1 | Z36 | -0.54 | -0.44 | -0.22 | -0.22 | -0.71 | -0.24 | -0.82 | -0.5 | -0.55 |
| ZB1 | Z42 | 4.55 | 2.27 | 1.12 | -0.37 | 2.01 | 3.43 | 2.61 | 2.72 | 2.79 |
| ZB1 | Z44 | -0.52 | -0.47 | -1.09 | -1.62 | -0.67 | -0.14 | -0.81 | -0.52 | -0.43 |
| ZB1 | Z48 | 1.15 | 0.17 | 0.04 | 0.41 | 0.32 | 0.42 | -0.09 | 0.18 | 0.29 |
| ZB1 | Z49 | -0.37 | -0.34 | 0.05 | 0.52 | -0.48 | -0.71 | -0.64 | -0.48 | -0.33 |
| ZB1 | Z50 | 1.49 | 0.59 | 0.12 | -0.5 | 0.56 | 1.18 | 4.19 | -0.48 | 0.46 |
| ZB1 | Z51 | -0.37 | -0.29 | 0.11 | 0.3 | -0.51 | -0.62 | -0.77 | -0.4 | -0.34 |
| ZB1 | Z54 | 3.5 | 6.24 | 5.7 | 6.1 | 0.34 | 4.92 | 1.46 | 4.16 | 6.04 |
| ZB1 | Z55 | 0.94 | 2.15 | 2.14 | 1.61 | -0.24 | 1.69 | 0.36 | 1.55 | 2.23 |
| ZB2 | Z30 | -0.06 | -0.25 | -0.2 | 0.3 | -0.65 | 0.14 | -0.79 | -0.56 | -0.22 |
| ZB2 | Z31 | -0.67 | -0.5 | -1.09 | 0.1 | -0.7 | -0.59 | -0.78 | -0.56 | -0.51 |
| ZB2 | Z33 | -0.49 | -0.46 | 0.19 | 0.25 | -0.69 | -0.33 | -0.79 | -0.54 | -0.45 |
| ZB2 | Z34 | -0.27 | -0.29 | 0.11 | 0.36 | -0.7 | -0.33 | -0.74 | -0.54 | -0.42 |
| ZB2 | Z37 | -0.41 | -0.42 | -0.15 | 0.27 | -0.64 | -0.3 | -0.7 | -0.49 | -0.39 |
| ZB2 | Z39 | -0.31 | -0.28 | -0.24 | -0.04 | -0.71 | -0.23 | -0.69 | -0.57 | -0.41 |
| ZB2 | Z40 | -0.28 | -0.14 | -0.39 | -0.3 | -0.69 | 0.09 | -0.75 | -0.51 | -0.14 |
| ZB2 | Z41 | 0.1 | 0.06 | -0.82 | 0.33 | -0.65 | 0.44 | -0.67 | -0.47 | -0.02 |
| ZB2 | Z43 | -0.15 | -0.26 | 0.01 | 0.03 | -0.6 | 0.17 | -0.72 | -0.49 | -0.27 |
| ZB2 | Z45 | 0.18 | -0.22 | 0.2 | 0.28 | -0.66 | 0.29 | -0.6 | 0.28 | -0.08 |
| ZB2 | Z46 | -0.7 | -0.4 | -0.23 | 0 | -0.71 | -0.59 | -0.87 | -0.38 | -0.52 |
| ZB2 | Z47 | -0.25 | -0.33 | -0.21 | -0.22 | -0.7 | -0.2 | -0.77 | -0.4 | -0.46 |
| ZB2 | Z53 | -0.1 | -0.27 | -0.23 | 0.09 | -0.26 | -0.44 | -0.42 | -0.41 | -0.34 |
| ZA | Z52 | -0.49 | -0.4 | 0.13 | 0.05 | 0 | -0.93 | -0.37 | -0.47 | -0.11 |
| ZA | Z56 | 0.72 | 0.14 | -1.07 | -1.53 | 2.2 | -0.26 | 1.75 | -0.43 | 0.13 |
| ZA | Z57 | -0.54 | -0.43 | -0.23 | -0.57 | -0.44 | -1.03 | -0.69 | -0.38 | -0.35 |
| ZA | Z58 | -0.43 | -0.45 | -1.07 | -0.81 | 1.11 | -0.91 | 0.46 | -0.43 | -0.44 |
| ZA | Z59 | -0.69 | -0.45 | -0.39 | -0.78 | 0.29 | -0.98 | -0.13 | -0.42 | -0.52 |
| ZA | Z60 | -0.52 | -0.33 | -0.22 | -0.57 | 0.23 | -0.94 | -0.18 | -0.41 | -0.47 |
| ZA | Z61 | -0.62 | -0.33 | -0.44 | -0.68 | -0.18 | -0.96 | -0.54 | -0.4 | -0.5 |
| ZA | Z62 | -0.89 | -0.57 | -0.44 | -0.58 | -0.16 | -0.88 | -0.57 | -0.45 | -0.56 |
| ZA | Z63 | -0.68 | -0.44 | -0.36 | -1.47 | -0.29 | -1.03 | -0.55 | -0.37 | -0.39 |
| ZA | Z64 | -0.35 | -0.1 | 0.18 | -0.09 | 0.04 | -0.79 | -0.32 | -0.43 | -0.39 |
| ZA | Z65 | 0.74 | 0.02 | -0.09 | -0.3 | 2.43 | -0.51 | 1.58 | -0.39 | -0.18 |
| ZA | Z66 | 0.97 | 0.06 | -1.06 | 0.18 | 2.09 | -0.54 | 1.22 | -0.39 | -0.07 |
| ZA | Z67 | 1.62 | 0.74 | 0.22 | -0.31 | 3.91 | 0.14 | 3.06 | -0.4 | 0.46 |
| ZA | Z68 | 1.32 | 0.76 | 1.16 | 0.4 | 3.28 | 0.16 | 2.25 | 0.29 | 0.69 |
| ZA | Z69 | 0.87 | 0.8 | -1.06 | -0.68 | 2.8 | 0.09 | 1.87 | -0.4 | 0.54 |
| ZA | Z70 | 0.12 | 0.19 | 0.93 | 0.35 | 0.93 | -0.64 | 0.21 | -0.38 | -0.12 |
| ZA | Z71 | -0.32 | -0.35 | -1.07 | -0.39 | 0.74 | -0.88 | 0.21 | -0.46 | -0.31 |
| ZA | Z72 | -0.33 | -0.43 | -0.25 | -0.69 | -0.45 | -0.68 | -0.56 | -0.37 | -0.52 |

ZA is *Z. armatum*; ZB1 samples, belonging to *Z. bungeanum*, are represented by pericarps from Hancheng; ZB2 samples, belonging to *Z. bungeanum*, are represented by pericarps from Fengxian; Others refers to the rest of the samples whose pericarps are red but do not belong to *Z. bungeanum*; C16:0 is palmitic acid; C18:0 is stearic acid; C20:0 is eicosanoic acid; C22:0 is behenic acid; C16:1 is palmitoleic acid; C18:1n9 is oleic acid; C18:1n13t is trans-13- oleic acid; C20:1 is cis-11-eicosenoic acid; C18:2 is linoleic acid; C18:3 is linolenic acid.

**Table S5** The effect of environmental factors on fatty acid variation for *Zanthoxylum* pericarps

| **Name** | **ZA** | | | | **ZB1** | | | | **ZB2** | | | |
| --- | --- | --- | --- | --- | --- | --- | --- | --- | --- | --- | --- | --- |
|  | **Explains %** | **Contribution %** | **pseudo-F** | ***p*** | **Explains %** | **Contribution %** | **pseudo-F** | ***p*** | **Explains %** | **Contribution %** | **pseudo-F** | ***p*** |
| Long | 2.1 | 2.1 | 2.1 | 0.078 | 1.2 | 1.4 | 1.0 | 0.378 |  |  |  |  |
| Lat |  |  |  |  | 0.9 | 1.0 | 0.7 | 0.520 | 5.3 | 5.3 | 1.0 | 0.392 |
| Alt | 1.6 | 1.6 | 1.8 | 0.174 | 1.5 | 1.7 | 1.2 | 0.268 | 6.4 | 6.4 | 1.1 | 0.398 |
| AtP |  |  |  |  | 1.0 | 1.2 | 0.8 | 0.486 | 9.8 | 9.8 | 2.2 | 0.176 |
| MT | 0.3 | 0.3 | <0.1 | 1.000 | 1.6 | 1.8 | 1.2 | 0.266 |  |  |  |  |
| MRH | 1.0 | 1.0 | 2.0 | 0.146 | 0.7 | 0.8 | 0.5 | 0.598 | 14.3 | 14.3 | 2.1 | 0.084 |
| MAP | 1.1 | 1.1 | 1.7 | 0.178 | 3.6 | 4.0 | 1.8 | 0.174 | 9.0 | 9.0 | 1.6 | 0.178 |
| pH | 0.5 | 0.5 | 1.7 | 0.296 | 1.1 | 1.2 | 0.9 | 0.402 | 11.5 | 11.5 | 1.8 | 0.132 |
| OM | 8.1 | 8.1 | 2.3 | 0.120 | 1.9 | 2.1 | 1.4 | 0.240 | 4.8 | 4.8 | 3.8 | 0.114 |
| N_t_ | 0.6 | 0.6 | 1.5 | 0.322 | 6.9 | 7.6 | 4.6 | 0.016* | 0.6 | 0.6 | <0.1 | 1.000 |
| P_t_ | 0.8 | 0.8 | 1.2 | 0.342 | 1.2 | 1.4 | 1.0 | 0.392 |  |  |  |  |
| K_t_ | 1.2 | 1.2 | 1.6 | 0.174 | 2.5 | 2.8 | 1.9 | 0.142 | 10.3 | 10.3 | 4.2 | 0.044* |
| N_a_ |  |  |  |  | 7.2 | 8.0 | 4.1 | 0.016* |  |  |  |  |
| P_a_ | 14.3 | 14.3 | 5.2 | 0.012* |  |  |  |  |  |  |  |  |
| K_a_ | 1.6 | 1.6 | 1.9 | 0.120 | 28.8 | 31.9 | 10.5 | 0.004** | 18.0 | 18.0 | 2.4 | 0.084 |
| Al | 3.9 | 3.9 | 3.6 | 0.012* | 4.4 | 4.9 | 2.1 | 0.104 | 2.0 | 2.0 | 3.5 | 0.240 |
| As | 0.9 | 0.9 | 1.3 | 0.328 | 1.2 | 1.3 | 0.9 | 0.422 |  |  |  |  |
| Cd | 15.5 | 15.5 | 11.9 | 0.002** | 17.5 | 19.4 | 8.2 | 0.032* | 8.0 | 8.0 | 1.3 | 0.304 |
| Pb | 39.0 | 39.0 | 10.2 | 0.002** | 2.5 | 2.7 | 2.0 | 0.138 |  |  |  |  |
| Mn | 7.4 | 7.4 | 3.1 | 0.048* | 1.1 | 1.2 | 0.9 | 0.394 |  |  |  |  |
| Ni |  |  |  |  | 3.5 | 3.9 | 2.5 | 0.064 |  |  |  |  |

ZA is *Z. armatum*; ZB1 samples, belonging to *Z. bungeanum*, are represented by pericarps from Hancheng; ZB2 samples, belonging to *Z. bungeanum*, are represented by pericarps from Fengxian; ** represents strong significant influence; * represents significant influence; Long is longitude; Lat is latitude; Alt is altitude; MT is mean temperature; MRH is mean relative humidity; MAP is mean annual precipitation; pH is power of hydrogen; OM is organic matter content in soil; N_t_ is total nitrogen content in soil; P_t_ is total phosphorus content in soil; K_t_ is total potassium content in soil; N_a_ is available nitrogen content in soil; P_a_ is available phosphorus content in soil; K_a_ is available potassium content in soil; Al is aluminium content in soil; As is arsenic content in soil; Cd is cadmium content in soil; Mn is manganese content in soil; Ni is nickel content in soi
