# Supplementary material for: Fatty Acid Profiling and Chemometric Analyses for Zanthoxylum Pericarps from Different Geographic Origin and Genotype
Source: Foods. 2020 Nov 16;9(11):1676. doi: 10.3390/foods9111676 (PMC7698129; doi:10.3390/foods9111676)
Supplement: Supplementary file 1 [file foods-09-01676-s001.zip › Supplementary Figures.doc]

Supplementary figures

Fatty Acid Profiling and Chemometric Analyses for Zanthoxylum Pericarps from Different Geographic Origin and Genotype

Yao Ma^1,2^, Jieyun Tian ^1,2^, Xiaona Wang ^1,2^, Chen Huang^1^, Mingjing Tian^1^, and Anzhi Wei^1,2*^

^1^	College of Forestry, Northwest A&F University; mayao277000@nwafu.edu.cn (Y. Ma), tianjieyun@nwafu.edu.cn (J. Tian), 17709590958@nwafu.edu.cn (X. Wang), hc19990513@nwafu.edu.cn (C. Huang), 15531971892@nwafu.edu.cn (M. Tian), and weianzhi@nwafu.edu.cn (A. Wei)

^2^	Research Centre for Engineering and Technology of *Zanthoxylum*, State Forestry Administration; mayao277000@nwafu.edu.cn (Y. Ma), tianjieyun@nwafu.edu.cn (J. Tian), 17709590958@nwafu.edu.cn (X. Wang), and weianzhi@nwafu.edu.cn (A. Wei)*****

Correspondence: weianzhi@126.com; Tel. 86-029-87082211; Yangling, 712100, China

Supplementary figures

**Fig.S1** The mean, median, standard deviation (SD), variance (CV), skewness coefficient, and p value for the Kolmogorov–Smirnov normality test of fatty acids (mg/g).

SFA is saturated fatty acid; MUFA is monounsaturated fatty acid; PUFA is polyunsaturated fatty acid; C16:0 is palmitic acid; C18:0 is stearic acid; C20:0 is eicosanoic acid; C22:0 is behenic acid; C16:1 is palmitoleic acid; C18:1n9 is oleic acid; C18:1n13t is trans-13- oleic acid; C20:1 is cis-11-eicosenoic acid; C18:2 is linoleic acid; C18:3 is linolenic acid.

**Fig.S2** The relative abundance of three fatty acids among different *Zanthoxylum* species.

The peaks without labels were identified as trace components. ZA is *Z. armatum*; ZB1 samples, belonging to *Z. bungeanum*, are represented by pericarps from Hancheng; ZB2 samples, belonging to *Z. bungeanum*, are represented by pericarps from Fengxian; Others refers to the rest of the samples whose pericarps are red but do not belong to *Z. bungeanum*; RT, retention time; C16:0 is palmitic acid; C18:0 is stearic acid; C20:0 is eicosanoic acid; C22:0 is behenic acid; C16:1 is palmitoleic acid; C18:1n9 is oleic acid; C18:1n13t is trans-13- oleic acid; C20:1 is cis-11-eicosenoic acid; C18:2 is linoleic acid; C18:3 is linolenic acid.

**Fig.S1** The mean, median, standard deviation (SD), variance (CV), skewness coefficient, and p value for the Kolmogorov–Smirnov normality test of fatty acids (mg/g).

SFA is saturated fatty acid; MUFA is monounsaturated fatty acid; PUFA is polyunsaturated fatty acid; C16:0 is palmitic acid; C18:0 is stearic acid; C20:0 is eicosanoic acid; C22:0 is behenic acid; C16:1 is palmitoleic acid; C18:1n9 is oleic acid; C18:1n13t is trans-13- oleic acid; C20:1 is cis-11-eicosenoic acid; C18:2 is linoleic acid; C18:3 is linolenic acid.

**Fig.S2** The relative abundance of three fatty acids among different *Zanthoxylum* species.

The peaks without labels were identified as trace components. ZA is *Z. armatum*; ZB1 samples, belonging to *Z. bungeanum*, are represented by pericarps from Hancheng; ZB2 samples, belonging to *Z. bungeanum*, are represented by pericarps from Fengxian; Others refers to the rest of the samples whose pericarps are red but do not belong to *Z. bungeanum*; RT, retention time; C16:0 is palmitic acid; C18:0 is stearic acid; C20:0 is eicosanoic acid; C22:0 is behenic acid; C16:1 is palmitoleic acid; C18:1n9 is oleic acid; C18:1n13t is trans-13- oleic acid; C20:1 is cis-11-eicosenoic acid; C18:2 is linoleic acid; C18:3 is linolenic acid.
